# Identifying the missing link in catalyst transfer polymerization

**DOI:** 10.1038/s41467-018-06324-9

**Published:** 2018-09-24

**Authors:** Weiying He, Brian O. Patrick, Pierre Kennepohl

**Affiliations:** Department of Chemistry, The University of British Columbia, 2036 Main Mall, Vancouver, BC V6T 1Z1 Canada

## Abstract

Nickel-catalyzed catalyst transfer polycondensation (CTP) of thiophenes is an efficient strategy for the controlled synthesis of polythiophenes. However, a detailed view of its reaction mechanism has remained elusive with unresolved questions regarding the geometry and bonding of critical Ni(0) thiophene intermediates. Herein, we provide experimental and computational evidence of structurally characterized square planar *η*^2^-Ni(0)–thiophene species and their relevance to the mechanism of CTP. These results confirm the viability of C,C-*η*^2^ bound intermediates in CTP of thiophenes, providing an electronic rationale for the stability of such species, and thus that such processes can proceed as living polymerizations. We further show that C,S-*κ*^*2*^ species may also be relevant in nickel-catalyzed CTP of thiophenes, providing new avenues for exploitation and optimization.

## Abstract (web summary)

The mechanism for nickel-catalyzed catalyst transfer polycondensation of thiophenes to polythiophenes. Here the authors show experimental and computational evidence of structurally characterized square planar *η*^2^-Ni(0)–thiophene species and their relevance to the mechanism of catalyst transfer polycondensation.

## Introduction

**Fig. 1:**
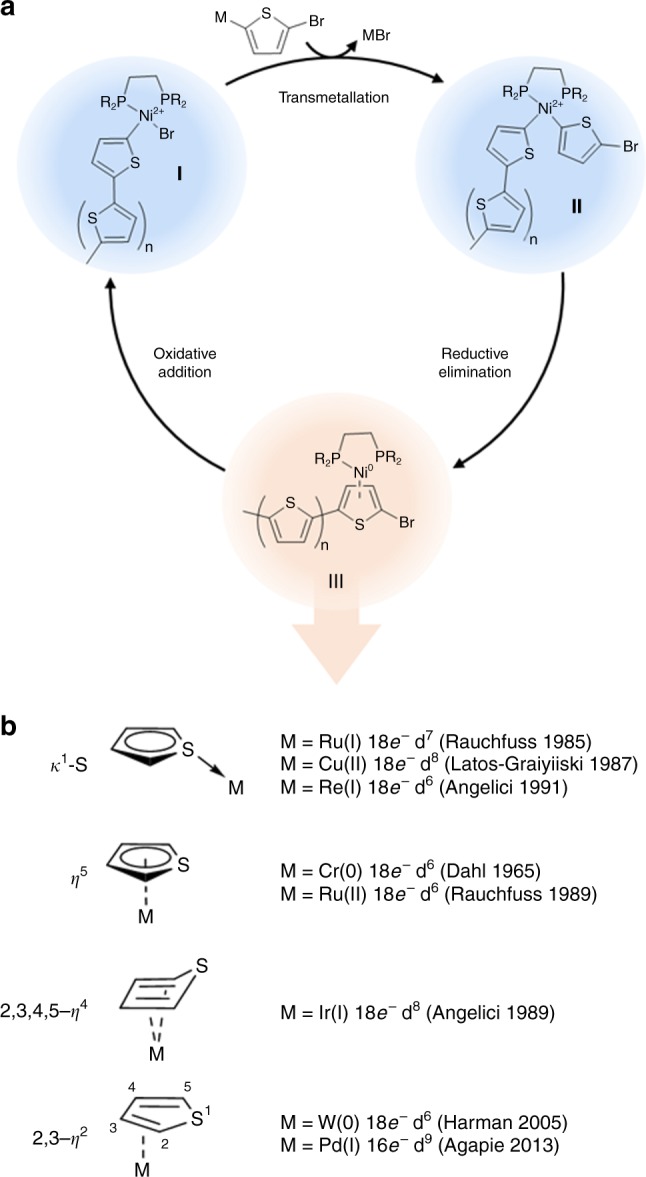
**a** Generally accepted mechanism for Ni-catalysed CTP. The Ni(II) species highlighted in blue (**I**, **II**) have been previously identified and characterized. By contrast, the putative Ni(0) species in red (**III**) has no precedent in the literature. **b** Structurally characterized thiophene binding modes from crystallographically defined metal–thiophene complexes

The much anticipated revolution from bulk to molecular electronics hinges upon efficient and controlled exploitation of appropriate molecular transistors, diodes, integrated circuits, and optoelectronic devices^1–3^. Polythiophenes have been widely explored as building blocks in both electronic and optical molecular devices due to their efficient electronic and thermal conductivity as well as their high quantum efficiencies^4–6^. Among current strategies for polythiophene synthesis, nickel-catalyzed catalyst transfer polycondensation (CTP) has proven particularly effective in the production of polythiophenes with high-molecular weights, regioregularity, low polydispersities, and end-group control^7–9^. The mechanism of thiophene CTP has attracted significant attention since it was first shown that it proceeded via living chain-growth polymerization^10–13^, which requires that the metal catalyst must remain attached to the nascent polymer chain throughout the process (Fig. 1a)^14^. Although stable Ni(II) intermediates have been characterized (**I** and **II**)^15,16^, the identity of the postulated Ni(0) intermediate (**III**) has remained elusive.

McNeil and coworkers have postulated that such Ni(0)–thiophene intermediates could, by analogy to known Ni(0)–arene complexes, be formulated as C,C*-η*^2^ Ni(0) structures^17^. Such structures are consistent with computational^18^ and experimental^19,20^ data, but conclusive structural evidence is lacking. However, computational studies indicate the feasibility of alternative Ni–thiophene isomers and the available spectroscopic data are not inconsistent with other potential structures. In fact, there exist several reasonable bonding modes in metal thiophenes (see Fig. 1b), including sulfur coordination (*κ*^1^-S)^21–23^ and a variety of π adducts (*η*^2^, *η*^4^, or *η*^5^)^24–27^. A direct analogy between arene and thiophene binding modes is, therefore potentially problematic as has been noted previously^23,28^. Only two structurally characterized complexes are known to adopt the proposed C,C-*η*^*2*^ binding motif in thiophene complexes: the Harman group identified a saturated 18*e*^−^ tungsten complex with a weakly bound C,C-*η*^*2*^ thiophene ligand^29^ and Agapie and coworkers isolated a unique palladium(I) dimer with a bridging cis-μ-η^*2*^*:η*^*2*^ thiophene^30^. Although these two reports demonstrate the feasibility of *C,C-η*^*2*^ binding for thiophene, they differ both structurally and electronically from the proposed CTP intermediates, and thus cannot directly address whether such bonding may be achieved in a low valent d^10^ system. For these reasons, elucidation of the geometric and electronic structure of species such as **III** remain an unsolved challenge until now^13,31,32^. Herein, we report the structure and properties of species that are directly relevant to the CTP process, providing an opportunity to evaluate the factors that allow for living polymerization of thiophenes.

## Results

### Factors relevant to isolating relevant intermediates

**Fig. 2:**
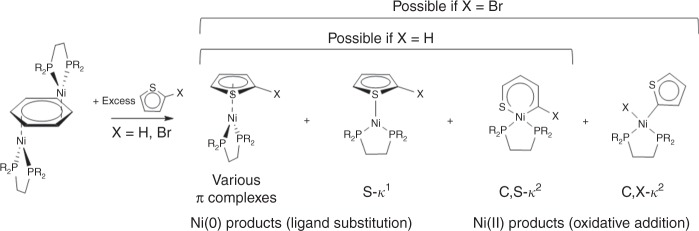
Possible products from reaction of Ni(0) arene precursor with excess thiophene reactants. In order to minimize the formation of unwanted oxidative addition products, thiophenes without a reactive 2-bromo substituent were chosen, leaving C–S insertion as the only other viable pathway for oxidative addition. Note that although the S-*κ*^1^ isomer is shown, it is not observed. Only one representative species of many possible isomers is shown for each of the possible outcomes

**Fig. 3:**
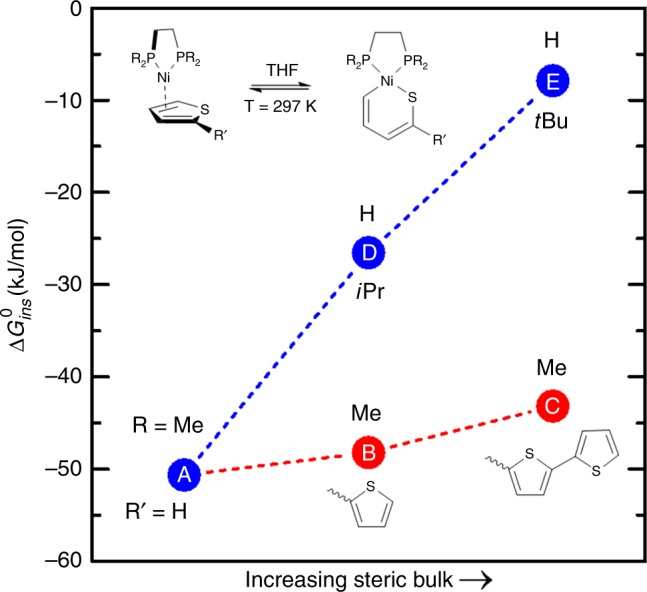
DFT-derived thermodynamics for C–S insertion from the *exo*-π adduct based on the B3LYP functional. Results obtained for the M06 and BP86 functionals yield the same trends as those shown here (see Supporting information for further detail). Increasing the steric bulk at the thiophene ligand (R’) results in a modest decrease in the preference for C–S insertion (A→B→C), whereas the effect is substantial when steric bulk is increased in the diphosphine ligand (R). The *x*-axis is a generic axis representing increasing steric bulk but it is not quantitative. Additional information is provided in Supplementary Table 7

We anticipated that a major challenge to successful isolation of Ni(0) thiophene π complexes would be to limit oxidative addition via cleavage of the C–X bond or the internal thiophene C–S bonds as shown in Fig. 2^33,34^. C–Br insertion can be avoided by choosing unsubstituted thiophene ligands and we anticipated that removing the bromine substituent would have a relatively minor effect on the electronic properties of the thiophenic ligand. This is supported by computational data (vide infra). Preventing C–S insertion, however, is a somewhat greater challenge. Given the more restricted geometry of the predicted C–S insertion product, we rationalized that increasing steric bulk near the metal centre could assist in minimizing C–S insertion. Density functional theory (DFT) calculations on the relative energies of the π complex relative to the C–S insertion product indicated a decreasing preference for C–S insertion on going from thiophene, bithiophene, and trithiophene (see Fig. 3A→B→C). The overall effect, however, is relatively small and ${\mathrm{\Delta }}G_{ins}^{DFT}$ remains significantly exergonic even with the trithiophene ligand (${\mathrm{\Delta }}G_{ins}^{B3LYP} < - 30 \hskip2pt {\mathrm{kJ/mol}}$) at room temperature. By contrast, steric bulk in the ancillary diphosphine ligand is calculated to have a surprisingly large effect on the overall energetics of this equilibrium (Fig. 3A→D→E); *t*-butyl substitution leads to a significant decrease in the preference for C–S insertion (${\mathrm{\Delta }}G_{ins}^{B3LYP} > - 10 \hskip2pt {\mathrm{kJ/mol}}$).

### Solution characterization of relevant species

**Fig. 4:**
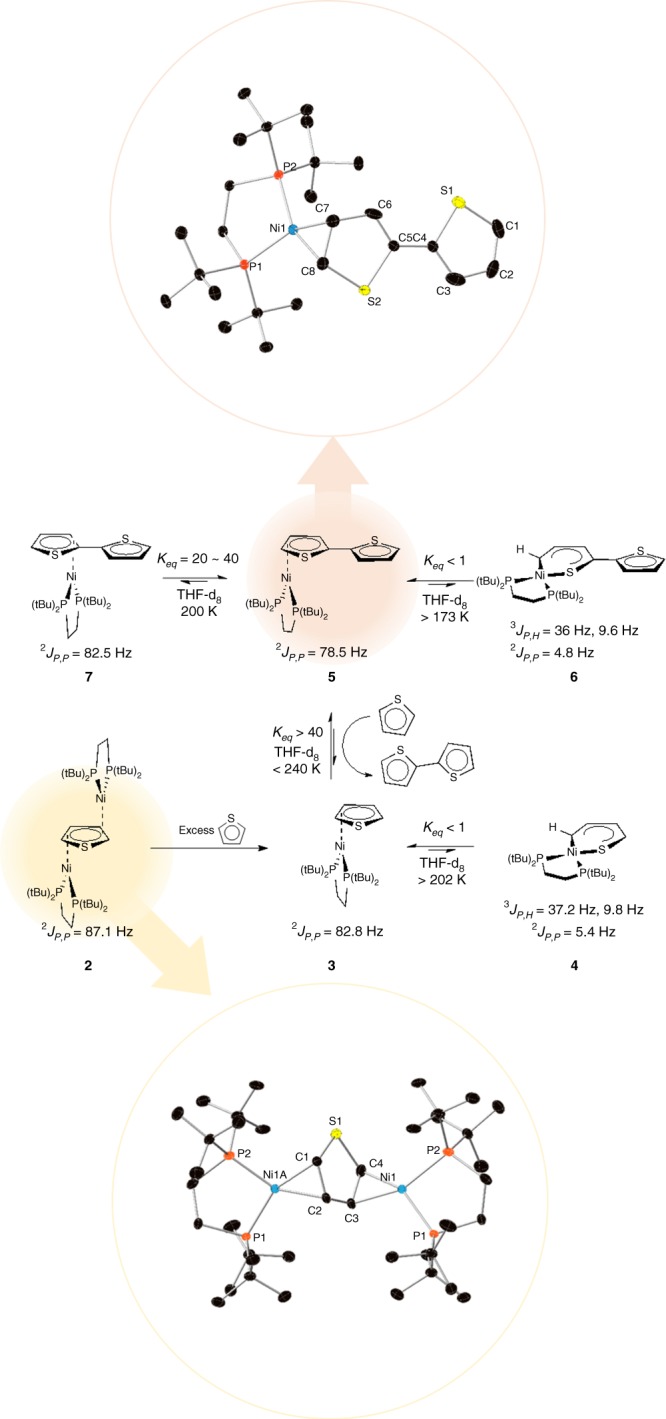
Experimentally determined equilibrium values (centre) and solid-state molecular structures of **2** (bottom) and **5** (top). All equilibrium data are given for reactions as written from left-to-right or bottom-to-top, as appropriate. The ORTEP diagrams of the crystallographically-determined coordinates are depicted with thermal ellipsoids at the 50% probability level. All hydrogen atoms are omitted for clarity. Selected bond lengths (Å) and angles (°) for 2: Ni1–P1, 2.190(2); Ni1–P2, 2.2283(16); Ni1–C3, 2.004(7); Ni1–C4, 1.953(5); C3–C4, 1.432(15); Ni1–Ni1i, 4.764(7) P1–Ni1–P2, 91.16(8); C3–Ni1–P1, 111.7(5); C3–Ni1–P2, 157.0(4); C4–Ni1–P1, 150.78(17); C4–Ni1–P2, 116.12(18); selected bond lengths (Å) and angles (°) for **5**: Ni–P1, 2.1831(5); Ni1–P2, 2.1970(5); Ni1–C7, 1.9985(18); Ni1–C8, 1.9570(18); C7–C8, 1.447(3); C5–C6, 1.338(6); C1–C2, 1.345(3); C3–C4,1.380(6); P1–Ni1–P2, 92.612(19); C7–Ni1–P1, 151.09(6); C7–Ni1–P2, 114.51(6); C8–Ni1–P1, 110.60(6); C8–Ni1–P2, 156.78(6); C8–Ni1–C7, 42.89(8). Structures have been deposited to the CCDC (CCDC1587316 for **2**, and CCDC1587317 for **5**). There is disorder observed in both the thiophene and bithiophene complexes, which does not affect the overall structural features of the complexes, see SI for refinement and details, Supplementary Figure 3

Inspired by Love and coworkers’ approach to the synthesis of Ni(0) π complexes^35^, we used a bulky diphosphine Ni(0) benzene dimer [(dtbpe)Ni]_2_(*μ-η*^*2*^:*η*^*2*^-C_6_H_6_) (**1**, dtbpe = 1,2-bis(di-*tert-*butyl) phosphinoethane) as a precursor from which to explore the formation of thiophene complexes in pentane at low temperature (~245 K). In the presence of a large excess of thiophene, an orange solid is obtained after in vacuo removal of solvent. The solid-state molecular structure of the recrystallized product (**2**), [(dtbpe)Ni]_2_(*trans*-*μ-η*^*2*^:*η*^*2*^-thiophene) indicates formation of a nickel(0) dimer with a bridging *μ-η*^2^:*η*^2^ thiophene ligand (Fig. 4, top), which is structurally analogous to the precursor benzene dimer. Solution NMR data of **2** are consistent with the solid-state molecular structure; notably, a large primary coupling between the inequivalent ^31^P nuclei (~87 Hz) implies a Ni(0) ground state configuration and additional splitting of the ^31^P signals are consistent with weaker *J*_*PP*_ coupling across the bridging thiophene ligand (5–8 Hz, see Supplementary Figure 11). Seeking to generate the target monomeric thiophene complex, we added a further excess of thiophene to **2** in THF at ~245 K. Under these conditions, an equilibrium mixture of the presumed mononuclear thiophene complex (**3**), (dtbpe)Ni(*η*^*2*^-thiophene) and the C–S insertion side-product (**4**), (dtbpe)Ni(S,C-*κ*^*2*^-thiophene) is obtained (see Supplementary Figure 15). The ^31^P nuclear magnetic resonance (NMR) data for **3** are similar to that of **2** although the observed *J*_PP_ is slightly lower in **3** (~82 Hz), which is consistent with stronger binding to the π ligand in the monomeric species. We have been unsuccessful in isolating and/or crystallizing **3**, presumably due to a significant enthalpic preference for **4** (See Supplementary Figures 1–2 and Supplementary Tables 1–2).

Our DFT calculations indicate that C–S insertion should be less favored in oligothiophenes as opposed to monothiophenes (Fig. 3) and thus we postulated that switching to bithiophene as a ligand might provide a better opportunity to isolate and characterize an analogous mononuclear complex (**5**, bithiophene), and suppress formation of the insertion product **6** (Fig. 4). This approach worked beautifully, allowing us to obtain diffraction quality single crystals of **5** and obtain its solid-state molecular structure (Fig. 4, bottom). This species is the only structurally characterized d^10^ metal–thiophene complex and exhibits an *exo*-C,C-*η*^*2*^ binding geometry. Importantly, the metal complex exhibits a square planar geometry, suggesting that π-backbonding plays a critical role in its stability^36^. As with the monothiophene complexes in solution, the **5** **=** **6** equilibrium can be followed by ^31^P{^1^H} NMR. As predicted from DFT, the preference for C–S insertion is decreased slightly in the bithiophene equilibrium (**5**–**6**) relative to the monothiophene. We attribute this to a modest enthalpic preference for the π complex in the bithiophene system as predicted computationally (Supplementary Figure 6 and Supplementary Table 7); available experimental data are also consistent with this hypothesis (Supplementary Table 1).

### Geometric and electronic structure of intermediate species

**Fig. 5:**
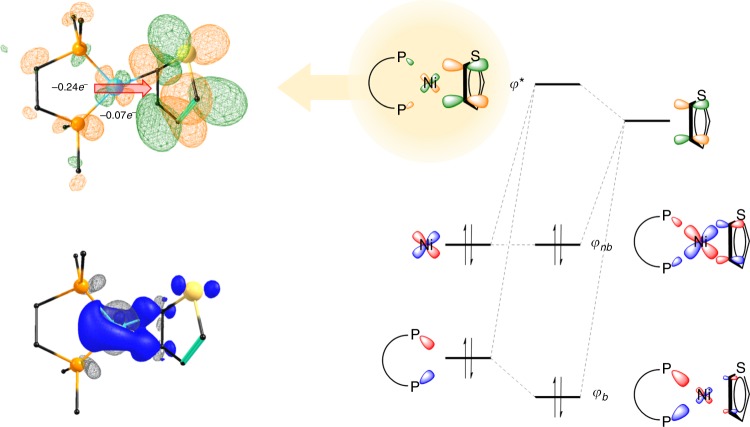
Dominant π-backbonding interaction between metal *bis*-phosphine fragment and the thiophene ligand. The interaction is formally a three-centre four-electron interaction between the three molecular fragments: the diphosphine ligand, the metal centre, and the thiophene ligand. This cooperative bonding interaction is most easily represented by looking at the formally antibonding orbital (−0.38 eV), which shows the degree of charge donation from both the metal centre (0.07*e*^−^) and the diphosphine ligand (0.24*e*^−^). An atoms-in-molecules (AIM) fragment analysis (bottom) additionally shows the overall charge redistribution from the diphosphine ligand and metal centre into the thiophene ligand (gray = decreased electron density, blue = increasing electron density), see more details in Supplementary Figures 7–8 and Supplementary Tables 8–9

The isolation and characterization of **5** provide an opportunity to directly probe the nature of a very close structural analog to nickel(0) CTP intermediates. The metric parameters for **5** indicate strong similarities to other Ni(0) π complexes. In addition, the metric and electronic properties of the complex are in good agreement with a DFT-optimized analog (RMSD ~8 pm, see Supplementary Figures 3–4, Supplementary Table 4). We have recently studied a broad range of well-defined nickel π complexes that are best described as square planar d^10^ complexes; this unusual bonding scheme derives from the dominance of backbonding from the metal to the π ligand through the same metal orbital (*dx*^2^–*y*^2^) that can receive electron density from the diphosphine ligand as shown in Fig. 5^36^. This situation formally creates a three-centre–four-electron (3c–4e)^37,38^ bond involving σ donation from the phosphines through to the *η*^2^ thiophene ligand via π backbonding from the nickel centre. Computationally, we estimate that almost 75% of the backdonation comes from the *trans*-phosphine ligands indicating a large cooperative binding effect through this 3c–4e interaction. The nature of this backbonding interaction was confirmed using Ni K-edge X-ray absorption spectroscopy (Supplementary Figure 9), which confirms that the metal centre remains electron rich and very Ni(0)-like. By comparison with other Ni π complexes, we note that the π acidity of the thiophene ligand is similar to that of ethylene in the analogous Ni(dtbpe)ethylene complex^36^. The delocalized 3c–4e interaction necessitates a nearly square planar geometry about the metal centre. It also points to the importance of a sterically encumbered but electron rich diphosphine ligand, which provides significant charge donation to strengthen the metal–thiophene bond. This “push–pull” effect is similar to that which has been observed as a key factor in the mechanism of Cytochrome P450 enzymes^39^, where a thiolate ligand provides charge donation across the metal centre to increase activation of a dioxygen-derived ligand. In this case, however, the push–pull effect leads to stabilization of the Ni(0) intermediates, which should affect ligand dissociation during catalysis. We therefore suggest that this effect is critical in ensuring living polymerization of thiophenes with nickel diphosphine catalysts.

The nature of bonding in the Ni(0) π complex is critical to the overall CTP mechanism as it represents the intermediate that is most weakly bound and thus most likely to release the polythiophene chain during turnover. Previous studies have indicated that the bond must be strong enough to prevent loss of the ligand but labile enough to allow for chain walking along the polythiophene backbone^14^. Intriguingly, At 200 K, we observe an additional species in solution (**7**) in equilibrium with the dominant **5** and **6** (see Fig. 4, middle). This species has similar spectroscopic properties to **5** and we assign this species to the *endo η*^2^_CC_ isomer. This is supported by computational results, where **5** is enthalpically favored over **7** due to additional steric constraints in the *endo* configuration (${\mathrm{\Delta }}H_{DFT}^0$= 6.3 kJ/mol, see Supplementary Tables 5–6 and Supplementary Figure 5). Based on available NMR data, we estimate *K*_*eq*_ = 20–40 at *T* = 200 K, suggesting a similar preference of 5–6 kJ/mol for the *exo* isomer. Furthermore, we observe kinetic trapping of **7** during synthesis of **5** at very low temperatures (<200 K). We are currently exploring the isomerization kinetics to provide insights into the dynamics of [Ni] walking along the polymer chain.

## Discussion

**Fig. 6:**
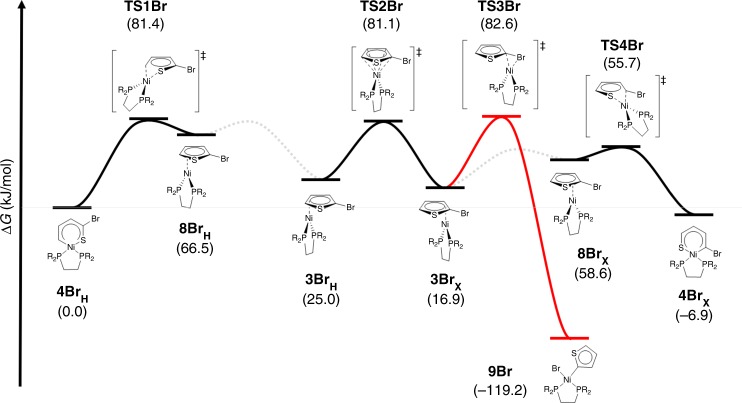
DFT calculated free energy of (dmpe)Ni(2-bromothiophene) ring transfer and oxidation addition in the gas phase (R = Me). Transition states connecting **3BrX** to **8BrX** and **3BrH** to **8BrH** have not been observed but coordinate scans indicate that these structural changes are associated with very low barriers

Our results demonstrate the viability of specific structural motifs as intermediates in the catalytic mechanism for CTP. Importantly, our results provide validation of DFT-derived geometric and electronic structure models for such species and increases confidence in the computational models. We, therefore, expanded our computational studies to include the catalytically relevant 2-bromo-substituted bithiophene complexes to explore the impact of halide substitution on the nature of the intermediates. In computational models, we find only minor differences upon inclusion of a 2-bromo substituent. As summarized in Table 1, the metric parameters about the metal centre are nearly identical in both cases (**3H** vs. **3Br**_**X**_). Notably, the **3Br** complex may adopt two different C,C-*η*^*2*^ isomers depending on whether the metal binds adjacent to (**3Br**_**X**_; C2,C3-*η*^2^ binding) or remote to (**3Br**_**H**_; C4,C5-*η*^*2*^ binding) the bromo substituent, these isomers are easily interconverted via a symmetric C,C,C,C-*η*^4^ transition state (**TS2Br** in Fig. 6)^18^. The calculated structure for **3Br**_**H**_ is very similar to **3H**, indicating that the bromo substituent has only a marginal impact over longer distances. The largest effect observed in **3Br**_**X**_ is a shift of the metal centre slightly towards the most electron deficient carbon (C2); this reflects greater asymmetry in the acceptor π* orbital of the 2-bromothiophene ligand. In addition, there is a very modest concomitant increase in the C = C bond distance, which is consistent with greater π backbonding in **3Br**_**X**_ due to bromo substitution.

**Table 1:** Comparison of bond distances (in pm) for C,C-*η*^2^ nickel complexes from crystallography (for the bithiophene analog 5) and from DFT calculations on model systems **3H**, **3BrX**, and **3BrH**.

bond	5^XRD^(Å)	3H^DFT^	3Br_X_^DFT^	3Br_H_^DFT^
Ni–P_1_	2.18(3)	2.19(0)	2.20(4)	2.19(7)
Ni–P_2_	2.19(7)	2.18(7)	2.19(9)	2.19(6)
Ni–C_1_	1.95(7)	2.00(0)	1.93(4)	1.98(4)
Ni–C_2_	1.99(9)	2.02(0)	2.02(0)	2.03(0)
C_1_–C_2_	1.44(7)	1.43(1)	1.43(6)	1.43(4)

Complete structural data for this series of (dmpe)Ni(thiophene) complexes is given in the appendix in the Supplementary Information. C_1_ is closest to the sulfur atom in the thiophene ligand and P_1_ is *cis*to C_1_. The diphosphine ligand in these calculations in the truncated dmpe = 1,2-bis(di-methyl) phosphinoethane ligand

Given that the computational method is robust for the ground state species, we expanded our investigation of the 2-bromosubstituted nickel complex to evaluate the overall energy profile for all possible isomers of this species. The resulting energy landscape for Ni(dmpe) binding to 2-bromothiophene is shown in Fig. 6. These data are in good agreement with experimental data that show that the C,C-*η*^2^ (**8Br**_**H**_) and C,S-*κ*^2^ (**4Br**_**H**_) species are in equilibrium, and that other possible isomers such as C,S-*η*^2^ isomers (**8Br**_**H**_ and **8Br**_**X**_) are much higher in energy (by >40 kJ/mol). Calculated barriers for isomerization are quite large due to the strong π-backbonding in the C,C-*η*^2^ structure. The barrier for C–Br oxidative addition (to form **9Br**) is similar to that for chain walking and also significantly larger than that for C–S oxidative addition near the bromo substituent (to form **4Br**_**X**_). We note that **4Br**_**X**_ is both the lowest energy species in the isomerization landscape (excluding formation of **9Br**) and that with the lowest barrier to formation. We, therefore, suggest that **4Br**_**X**_ and similar species formed during polymerization may serve as off-cycle intermediates during turnover. Such species are clearly important in hydrodesulfurization^33,34,40^, and we are currently exploring their relevance in CTP.

**Fig. 7:**
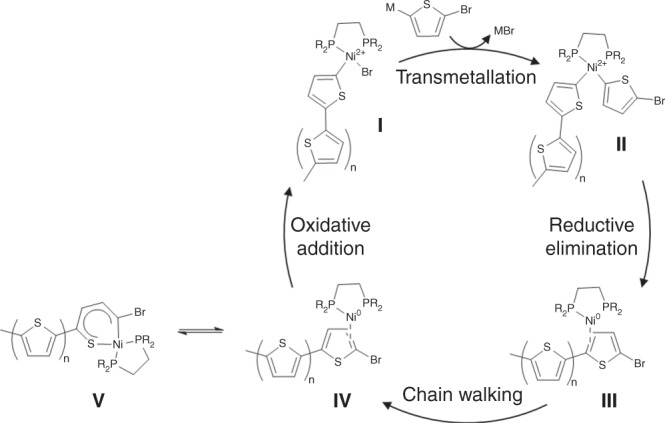
Proposed mechanism for CTP as supported by crystallographic and NMR spectroscopic evidence from this work. A close analog **7** to **III** is observed by low temperature 31P{H} NMR, and **5** is a model for **IV** has characterized via crystallographic and spectroscopic data. Preliminary computational data suggest the potential relevance of species such as V as off-cycle resting states

The minor differences observed in our computational studies of the unsubstituted and 2-bromosubstituted Ni(0) C,C-*η*^2^ thiophene complexes is strong evidence that **5** is highly relevant as a model for the actual intermediates in nickel-catalyzed CTP. In addition, our work supports the proposal that both the higher energy *endo* isomer (identified by NMR) and the *exo*-C,S-*κ*^2^ isomer (from DFT) are also relevant to the catalytic reaction mechanism. In fact, these results strongly support the idea that catalysis must proceed through both Ni(0) species as has been previously proposed^13,17^. C_sp2_–C_sp2_ coupling via reductive elimination necessarily generates an *endo-η*^2^_CC_ species (**III**, Fig. 7), which must undergo ring walking to the more stable *exo-η*^2^_CC_ species (**IV**, Fig. 7). The relative energies of these isomers provides a bias toward the catalytically competent *exo-η*^2^_CC_. We further propose that the off-cycle C,S-*κ*^2^ species may be important in the overall process by providing an off-cycle resting state prior to turnover limiting C–Br oxidative addition. We are currently exploring the details of migratory processes across the polymer chains as well as the impact of off-cycle C–S insertion to establish the factors that control this important step in the overall CTP process.

Our studies on the unsupported *η*^2^_CC_-bound Ni(0) thiophene complex confirm the overall structure of previously proposed intermediates in CTP and yield new insights into the mechanism of action and the importance of the diphosphine ligand in ensuring strong metal–thiophene bonding via a dominant push–pull π-backbonding interaction. Living polymerization conditions may occur due to this surprisingly strong bond between the d^10^ metal centre and the growing polymer chain. A preference for the catalytically competent *exo-η*^2^_CC_ isomer is also observed. These conclusions provide clarity into the mechanism of catalyst transfer polycondensation and hence provide the basis for further work on improving catalyst performance in the efficient synthesis of an important building block for molecular electronics.

## Methods

### Synthetic methods

Unless stated otherwise, all reactions were performed in a glovebox under an atmosphere of pure nitrogen using standard Schlenk techniques. Anhydrous pentanes, toluene, diethyl ether, and tetrahydrofuran were purchased from Aldrich, sparged with dinitrogen, and dried further by passage through towers containing activated alumina and molecular sieves. THF-d_6_, C_6_D_6_, and toluene-d_8_ were purchased from Aldrich and dried over sodium/benzophenone before being distilled and degassed by three freeze–pump–thaw cycles. Thiophene was purchased from Aldrich, were dried over activated 4 Å molecular sieves, distilled and degassed by three freeze–pump–thaw cycles. Bithiophene was purchased from Matrix and degassed by vacuum. Complex **1** was prepared according to the literature procedures. NMR spectra were recorded on 300, 400 MHz spectrometers and are referenced to residual protio solvent (7.16 ppm for C_6_D_5_H, 2.08 ppm for the methyl resonance of toluene-d_8_, and 5.32 ppm for CDHCl_2_) for 1H NMR spectroscopy, solvent peaks (128.06 ppm for C_6_D_6_, 53.84 ppm for CD_2_Cl_2_, and 20.43 ppm for the methyl resonance of toluene-d_8_) for ^13^C NMR spectroscopy. ^31^P NMR spectra were referenced to 85% H_3_PO_4_ at 0 ppm. Mass spectra and elemental analyses were performed by the microanalytic services at the Department of Chemistry of the University of British Columbia. Details of specific synthetic procedures are included as Supplementary Methods; relevant NMR characterization of complexes is given in Supplementary Figures 10–22.

### Crystallographic methods

All measurements were made on a Bruker APEX DUO diffractometer with a TRIUMPH curved-crystal monochromator with Mo-Kα radiation. The data were collected at a temperature of −183.0 ± 0.1 ^o^C. Data were collected in a of ϕ and ω 0.5^o^ oscillations using 20.0 second exposures. The crystal-to-detector distance was 40.14 mm. Data were collected and integrated using the Bruker SAINT software package. Data were corrected for absorption effects using the multi-scan technique (SADABS). The data were corrected for Lorentz and polarization effects. The structure was solved by direct methods. In refinement of structure 5, the Ni–thiophene–Ni fragment is disordered and was modeled in two orientations with equal proportions. in refinement of structure **5**. The material crystallizes with the bis-thiophene disorder in two orientations. The two orientations are related by a 180° rotation about an axis parallel to the C4–C5 bond. All nonhydrogen atoms were refined anisotropically. All hydrogen atoms were placed in calculated positions. The weighting scheme was based on counting statistics. Neutral atom scattering factors were taken from Cromer and Waber. Anomalous dispersion effects were included in *F*_calc_; the values for *f*' and *f*'' were those of Creagh and McAuley. The values for the mass attenuation coefficients are those of Creagh and Hubbell. All refinements were performed using the SHELXL-2016 via the OLEX2 interface. Additional crystallographic details are given in Supplementary Table 3.

### Computational methods

DFT calculations initial geometries for all molecules were obtained from crystallographic coordinates (where available) or constructed from standard models. Geometry optimizations and numerical frequency calculations were performed using version 3.0.3 of the ORCA computational chemistry package. Molecular geometries were optimized using the B3LYP functional and all electron basis sets (def_2_-TZVP) for all atoms. Optimized geometry coordinates are given in Supplementary Table 11. Explorations of the relative energies of different nickel thiophene isomers (see Fig. 6) were performed with a truncated diphosphine ligand (R = Me) rather than the complete *tert*-butyl ligand to manage computational expense. Statistical mechanics calculations of entropic and thermal effects were performed using the rigid rotor and harmonic oscillator approximations at 298.15 K and 1 atm. Potential energy surface scan was applied to find the potential intermediates and transition states geometries, and intrinsic reaction coordinates was calculated to confirm the connection between the transition state and reactants/products and calculation determined intermediates. Accurate single point energies were calculated with solvation model based on density solvent effect using M06 functional. Computational efficiency was improved by applying the RI approximation (RIJCOSX) for the hybrid functional. All calculations were performed with integration grid (ORCA Grid4). XAS TD-DFT calculation were performed with a dense intergration grid (Gird6) for better implementing Scalar relativisitic effects by using ZORA corrections, and reduced by using MOanlayzer software. NBO calculations were calculated with Gaussian 09 program package, AIM^41^ and CDA^42^ calculation were performed in Multiwfn software by using NBO outputs using B3LYP/def_2_-TZVP level. All calculations were run on the UBC Chemistry Abacus cluster and on the Westgrid GREX cluster.

### X-ray absorption spectroscopy

All the X-ray absorption spectroscopy (XAS) samples except Ni(dtbpe)bithiophene complex were analyzed as solids under anaerobic conditions and diluted in boron nitride (20–50% by weight). Ni(dtpe)bithiophene complex was prepared in dry toluene solvent and treated with extra bithiophene at RT to avoid the formation of S–C insertion byproduct Ni(dtbpe)(*κ*^2^-C,S-bithiophene) and quickly frozen under liquid nitrogen environment. XAS Ni K-edges were acquired at the SSRL beamline 7-3, which is equipped with a Si(220) *ϕ* = 90° double crystal monochromator, a 9 keV cutoff mirror, and a He cryostat (at 20 K). Data were collected using a Canberra 30-element Ge solid-state detector with a 3 mm Co filter. Data averaging and energy calibration were performed using SixPack, The AUTOBK algorithm available in the Athena software package was employed for data reduction and normalization. [Ni(dtbpe)_]2_-arene complex and Ni(dtbpe)Cl_2_ were used as reference to evaluate the oxidation state. Specifics of XAS fitting parameters are given in Supplementary Table 10.

## Electronic supplementary material

Supplementary Information

## Data Availability

The authors declare that the main data supporting the findings of this study are available within the article and its Supplementary Information files. Crystallographic data are available through the Cambridge Crystallographic Data Centre: CCDC identifiers are CCDC1587316 (complex **2**) and CCDC1587317 (complex **5**). Extra data are available from the corresponding author upon request.
