# Supplementary material for: Identifying the missing link in catalyst transfer polymerization
Source: Nat Commun. 2018 Sep 24;9:3866. doi: 10.1038/s41467-018-06324-9 (PMC6155128; doi:10.1038/s41467-018-06324-9)
Supplement: Supplementary file 1 — Supplementary Information [file 41467_2018_6324_MOESM1_ESM.pdf]

Supplementary Information

Identifying the missing link in catalyst transfer polymerization of thiophenes

Supplementary Methods

General Considerations

Unless stated otherwise, all reactions were performed in a glovebox under inert N_2 atmosphere. Anhydrous pentanes, toluene, diethyl ether, and tetrahydrofuran were purchased from Aldrich, sparged with dinitrogen, and dried further by passage through towers containing activated alumina and molecular sieves. THF-d_6, C_6D_6 and toluene-d_8 were purchased from Aldrich and dried over sodium/benzophenone before being distilled and degassed by three freeze-pump-thaw cycles. Thiophene was purchased from Aldrich, dried over activated 4Å molecular sieves, distilled and degassed by three freeze-pump-thaw cycles. Bithiophene was purchased from Matrix and degassed by vacuum. Complex **1** was prepared according to the literature procedures[1]. NMR spectra were recorded on 300, 400 MHz spectrometers and are referenced to residual protio solvent (7.16 ppm for C_6D_5H, 2.08 ppm for the methyl resonance of toluene-d_8, 5.32 ppm for $CDHCl_2$) for 1H NMR spectroscopy, solvent peaks (128.06 ppm for C_6D_6, 53.84 ppm for CD_2Cl_2, 20.43 ppm for the methyl resonance of toluene-d_8) for ^{13}C NMR spectroscopy. ^{31}P NMR spectra were referenced to 85 % H_3PO_4 at 0 ppm. Mass spectra and elemental analyses were performed by the microanalytic services at the Department of Chemistry of the University of British Columbia, Vancouver.

Synthesis and Isolation of 2

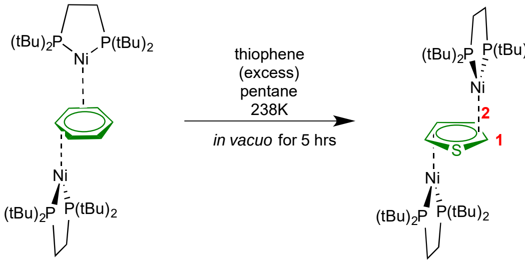

In a nitrogen-filled dry glovebox, a solution of **1** (38 mg, 0.0459mmol) in pentane was treated with thiophene (10 mg, 0.1189mmol) and reacted for 10 mins at -35 °C, resulting in a colour change of the solution to orange. The solvent was evaporated *in vacuo* for 5 hours and the crude product was extracted with cool pentanes stored at -35 °C, filtered through glass fiber to give an orange filtrate and stored at -35 °C. Yield 33.8mg (0.04051 mmol, 88.3 %), X-ray quality orange crystals of 2 were grown by slow evaporation of a saturated Et_2O/pentanes (1:1 ratio) solution in an open 1 dram vial sealed in a 5 dram vial over 2 days. Analytical data for 2. ^1H NMR (400 MHz, THF-d_8 , -65 °C) δ 4.12 (t, J = 7.8, 6.1 Hz, 2H, H1), δ 3.56 (dd, J = 13.4, 5.3 Hz, 2H, H2), δ 1.95 – 1.79 (m, 8H , PC**H₂**C**H₂**P), δ 1.50 – 1.39 (m, 8H, PC**H₂**C**H₂**P), δ 1.30 (dd, J = 11.4, 6.8 Hz, 18H , C(C**H**$_3$)$_3$), δ 1.22 (dd, J = 24.3, 10.8 Hz, 18H, C(C**H**$_3$)$_3$). $^{31}P\{^1H\}$ NMR (162 MHz, THF-d_8,-50 °C) δ 84.36 (d[AB], ddd, $^2J_{P,P}$ = 87.1, $^5J_{P,P}$=10.1, 6.3 Hz), 77.17 (d[AB], ddd, $^2J_{P,P}$ = 87.1, $^5J_{P,P}$=10.1, 6.3 Hz). $^{31}P\{^1H\}$ NMR (162 MHz, THF-d_8, 25 °C) δ 86.60 – 83.25 (m), δ 80.24 – 77.17 (m). $^{13}C\{^1H\}$ NMR (101 MHz, THF-d_8,-50 °C) δ 64.59 (d, $J_{C,P}$ = 18.0 Hz, C1), δ 50.30 (d, $J_{C,P}$ = 25.3 Hz, **C**2), δ 34.70 – 34.36 (m, **C**(CH$_3$)$_3$), δ 34.07 – 33.70 (m, **C**(CH$_3$)$_3$), δ 30.53 (d, J = 7.5 Hz, C(**C**H$_3$)$_3$) δ 30.14 (dd, J = 17.4, 6.5 Hz, C(**C**H$_3$)$_3$), δ 29.66 (d, J = 6.9 Hz, C(**C**H$_3$)$_3$) 22.88 (dd, J = 21.1, 12.1 Hz, P**C**H$_2$**C**H$_2$P). Anal. Calcd for $C_{39}H_{82}Ni_2P_4S$(%): C, 56.82; H: 10.03. Found: C: 56.03; H: 10.36. LRMS (MALDI) 838.6 [M+].

Formation of 3

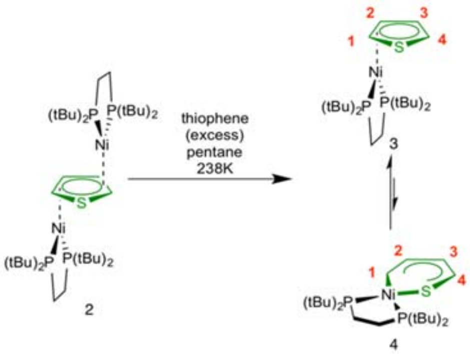

In a nitrogen-filled dry glovebox, A solution of **2** (20mg, 0.0435mmol) in 1.5 mL THF-d_8 was treated with thiophene (30 mg, 0.35mmol); the resulting orange solution was placed in a J-Young NMR tube at RT. The sample was place in an NMR probe and cooled to -65 °C degree. Analytical data for complex **3**. ^{1}H NMR (400 MHz, THF-d_8 , -65 °C) δ 6.35 (br, s, 1H, H4), δ 5.89 (br, s, 1H, H3), 4.52 (br, s, 1H, H2), 4.22 (br, s, 1H, H1), δ 1.39 – 1.16 (m, overlapped peaks of C(C**H**$_3$)$_3$ and PC**H$_2$**C**H$_2$**P). ^{31}P{^{1}H} NMR (162 MHz, THF-d_8 -60 °C) δ 79.33 (d[AB] d, $J_{P,P}$ = 82.8 Hz), 76.52 (d[AB] d, $J_{P,P}$ = 82.8 Hz). ^{13}C{^{1}H} NMR (101 MHz, THF-*d*$_8$,-50 °C) δ 126.70 (s, C4), δ 125.07 (s,C3), δ 65.61 (s, C2), δ 64.58(s, C2), δ 35.88 – 35.28 (m, **C**(CH$_3$)$_3$), δ 35.05 (d, J = 8.4 Hz, **C**(CH$_3$)$_3$), δ 31.23 – 30.75 (m, C(**C**H$_3$)$_3$), δ 29.09 (d, J = 13.6 Hz, C(**C**H$_3$)$_3$), δ 23.33 – 22.43 (overlapped with other peaks, P**C**H$_2$**C**H$_2$P).

Formation of 4

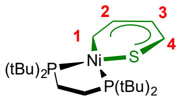

Analytical data for **4**. Clear and sharp peaks of **4** were found at -30 °C by ^{31}P{^{1}H} NMR spectroscopy, where in a 2:5 ratio over 3, and the amount of **4** got increased as the tempature went lower. The equilibrium has been confirmed as the ratio hasn't changed over 70mins. Analytical data for **4**. ^{1}H NMR (400 MHz, THF-d_8 , -65 °C) δ 8.02 (td, J =37.2, 9.8 Hz, 1H, H1), δ 6.72 (dd, J = 12.1, 10.5 Hz, 10H), δ 6.61 (m, J = 17.2, 7.9 Hz, 1H), δ 6.53 (dd, J = 15.0, 7.6, Hz, 1H), δ 2.12 (dd, J = 16.3, 7.8 Hz, 2H, PC**H$_2$**C**H$_2$**P), δ 1.94 (dd, J = 8.2, 7.7 Hz, 2H, PC**H$_2$**C**H$_2$**P), δ 1.47 (dd, J = 11.8, 7.6 Hz, 36H, C(C**H**$_3$)$_3$). ^{31}P{^{1}H} NMR (162 MHz, THF-d_8 -60 °C) δ 73.44 (d[AB] d, $^{2}J_{P,P}$ = 5.4 Hz), δ 67.03 (d[AB] d, $^{2}J_{P,P}$ = 5.4 Hz). ^{13}C{^{1}H} NMR (101 MHz, THF-*d*$_8$,-50 °C) δ 128.78 (s, C1), δ 128.18 (s, C2), δ 128.05 (s, C4), δ 125.18 (s, C3), δ 34.86 – 34.68 (m, **C**(CH$_3$)$_3$), δ 29.17 – 28.99 (m, C(**C**H$_3$)$_3$), δ 23.33 – 22.43 (overlapped with other peaks, P**C**H$_2$**C**H$_2$P).

Formation of 7

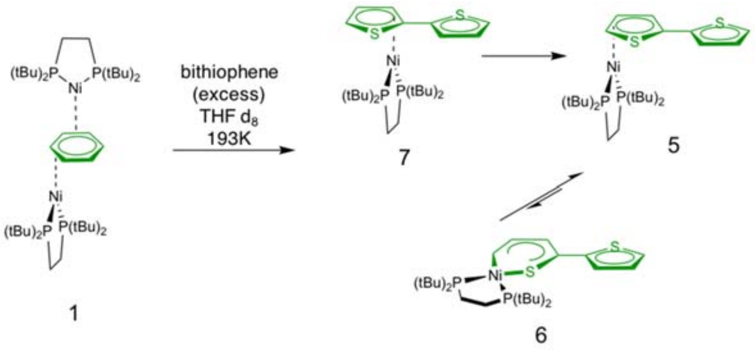

Complex **1** (20 mg, 0.0239 mmol) was dissolved in 1 mL of THF-d_8. This red-orange solution was transferred to a screw-cap NMR tube and frozen in liquid nitrogen. To the top of this frozen solution was added a solution of bithiophene (8 mg, 0.0481 mmol, 2 equiv) in THF-d_8 (0.2 mL). The tube was then frozen in liquid nitrogen before being quickly placed in an NMR spectrometer pre-cooled to -80 °C. sample was only analyzed by ^{31}P NMR spectrum, due to a short time existence of a small amount of **7**. ^{31}P{^{1}H} NMR (162 MHz, THF-d_8 -60 °C) δ 80.84 (d[AB] d, J = 82.5 Hz), δ 77.83 (d[AB], overlapped with other peaks).

Synthesis and Isolation of 5

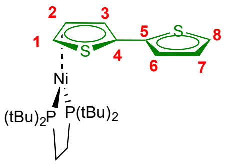

In a nitrogen-filled dry glovebox, a solution of **1** (10mg, 0.0119 mmol) in cool pentanes (-35 °C) was treated with a solution of bithiophene (8 mg, 0.0481 mmol) in cool pentanes (-35 °C) and reacted for 1h at -35 °C, resulting in a color change from fresh red into carrot orange. Saturated pentanes solution was placed in an open 1 dram vial sealed in a 5 dram vial and stored at -35 °C. After evaporating extra pentanes *in vacuo.* X-ray quality carrot orange crystals of 3 could be collected over several days. Analytical data for monomer-bithiophene-S3. ^{1}H NMR (400 MHz, THF-d_8 , -45 °C), δ 7.09 (d, J = 4.1 Hz, 1H, H_6), δ 6.86 (t, J = 4.2 Hz, 1H, H_7), δ 6.74 (d, J = 3.1 Hz, 1H, H_8), δ 6.64 (br, s, 1H, H_3), δ 4.42 (dd, J = 13.8, 4.4 Hz, 1H, H_2), δ 4.35 – 4.26 (m, 1H, H_1), δ 1.85 (dd, J = 21.2, 6.6 Hz, 2H, $P\mathbf{CH_2CH_2}P$), δ 1.63 – 1.56 (m, 2H, $P\mathbf{CH_2CH_2}P$), δ 1.32 (d, J = 5.3 Hz, 9H, $C(\mathbf{CH_3})_3$), δ 1.29 (d, J = 4.7 Hz, 9H, $C(\mathbf{CH_3})_3$), δ 1.25 (d, J = 10.5 Hz, 9H, $C(\mathbf{CH_3})_3$), δ 1.17 (d, J = 11.5 Hz, 9H, $C(\mathbf{CH_3})_3$). ^{13}C NMR (101 MHz, THF-d_8, -50 °C) δ 140.65 (d, J = 3.1 Hz, C_5), δ 127.11 (s, C_6), δ 122.40 (s, C_7), δ 120.63 (s, C_8), δ 120.62 (d, J = 20.2 Hz, C_4), δ 115.91 (s, C_3), δ 59.60 (m, J = 16.8 Hz, C_2), δ 50.89 (m, J = 24.2 Hz, C_1), δ 35.86 – 35.50 (m , $\mathbf{C}(CH_3)_3$), δ 35.34 (t, J = 6.1 Hz, $\mathbf{C}(CH_3)_3$), δ 34.79 (dd, J = 8.3, 3.6 Hz, $\mathbf{C}(CH_3)_3$), δ 33.94 – 33.40 (m, $C(\mathbf{CH_3})_3$), δ 30.18 (t, J = 7.2 Hz, $C(\mathbf{CH_3})_3$), δ 29.78 – 29.26 (m, $P\mathbf{C}H_2\mathbf{C}H_2P$). ^{31}P{^{1}H} NMR (162 MHz, THF-d_8 -70 °C) δ 81.66 (d[AB] d, $^{2}J_{P,P}$ = 78.4 Hz) δ 78.98 (d[AB] d, $^{2}J_{P,P}$ = 78.5 Hz).Anal. Calcd for $C_{26}H_{46}NiP_2S_2$(%): C, 57.47; H: 8.53. Found: C: 56.80; H: 9.11. LRMS (MALDI) 543.3 [M+].

Formation of 6

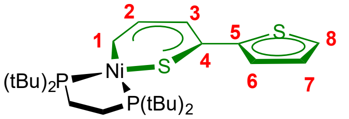

6 was found at -35 °C by ^{31}P{^{1}H} NMR spectroscopy, where in a 1:4 ratio over **6**. The equilibrium has been confirmed as the ratio hasn't changed over 70mins. ^{1}H NMR (400 MHz, THF-d_8 , -45 °C), δ 7.99 (dt, J = 36.0, 9.6 Hz, 1H), δ 7.30 (d, J = 3.1 Hz, 1H), δ 7.15 (d, J = 5.0 Hz, 1H), δ 7.07 δ (d, J = 3.7 Hz, 1H), δ 6.98 (d, J = 6.8 Hz, 1H), δ 6.93 (t, J = 4.3, 3.0 Hz, 1H), δ 2.15 (dq, J = 16.4, 8.1, 7.5 Hz, 2 H, $P\mathbf{CH_2CH_2}P$), δ 2.05 – 1.94 (m, 2 H, $P\mathbf{CH_2CH_2}P$), δ 1.50 (dd, J = 18.9, 11.9 Hz, 36H, $C(\mathbf{CH_3})_3$). ^{31}P{^{1}H} NMR (162 MHz, THF-d_8,-60 °C), δ 75.29 (d[AB] d, $^{2}J_{P,P}$ = 5.0 Hz), 68.88 (d[AB], d, $^{2}J_{P,P}$ = 4.8 Hz). ^{13}C{^{1}H} NMR (101 MHz, THF-d_8, -70 °C), δ 149.92(s), δ 135.79(s), δ 129.01 – 128.74 (m), 127.85 – 127.47 (m) , 128.17 (s), 124.25 (s), 123.76 (s), 122.61 (dt, J = 8.5, 4.8 Hz), 35.81 (d, J = 3.9 Hz, $\mathbf{C}(CH_3)_3$), 35.04 – 34.62 (m, $\mathbf{C}(CH_3)_3$), δ 33.86 – 33.55 (m, $\mathbf{C}(CH_3)_3$), δ 30.23 (d, J = 7.4 Hz, $C(\mathbf{C}H_3)_3$, overlapped with peaks of **6**), δ 23.09 (s, $P\mathbf{C}H_2\mathbf{C}H_2P$).

Variable-Temperature NMR Experiment

Equation 1 (an adaption of the Van't Hoff equation) allows for determination of $\Delta H^0{}_{INS}$ and $\Delta S^0{}_{INS}$ by plotting experimental data for the equilibrium Ni(dpte)-thiophene π adduct concentration $[M]_{add}$ and insertion product $[M]_{ins}$ in THF-d_8 at various temperatures (T). As the insertion equilibrium constant between two isomers of Ni-thiophene/ Ni-bithiophene complexes directly equals to the intensity ratio of two complexes' ^{31}P NMR peak integrity (Keq= $[M]_{ins}$ / $[M]_{add}$ = n_{ins} / n_{add}, the volume of the solution has no effect),

$$\ln\left[K_{eq}\right] = \frac{\Delta H^0_{\mathrm{INS}}}{RT} - \frac{\Delta S^0_{\mathrm{INS}}}{R} \qquad (1)$$

we can obtain experimental $\Delta H^0{}_{INS}$ and $\Delta S^0{}_{INS}$ via integration of ^{31}P signals assigned to the Ni π adduct and C-S insertion complexes, respectively. The formation of dimeric species was suppressed by addition of a large excess of the thiophene ligand. This large excess does not affect the monomer equilibrium.

$$T_{eq} = T_{(K_{eq}=1)} = \frac{\Delta H^0_{\mathrm{INS}}}{\Delta S^0_{\mathrm{INS}}} \qquad (2)$$

In binary equilibrium systems where the enthalpy (ΔH) and entropy (ΔS) have the same sign, there exists a temperature where $K_{eq} = 1$. This temperature is easily defined as shown in equation 2. This value is particularly relevant in this case as it represents the temperature at which the equilibrium shifts from preferring reactants to products (in an analogous fashion to an equilibrium constant. T_{eq} is easily obtained from a Van't Hoff analysis (equation 1).

X-ray diffratction of Complex 2.

An orange irregular crystal of $C_{40}H_{84}Ni_2P_4S$ having approximate dimensions of 0.06 x 0.10 x 0.13 mm was mounted on a cryo-loop. All measurements were made on a Bruker APEX DUO diffractometer with a TRIUMPH curved-crystal monochromator with Mo-Kα radiation. The data were collected at a temperature of -183.0 ± 0.1°C to a maximum 2θ value of 52.8°. Data were collected in a series of ϕ and ω scans in 0.5° oscillations using 20.0-second exposures. The crystal-to-detector distance was 40.14 mm. Of the 39334 reflections that were collected, 4651 were unique (R_{int} = 0.062); equivalent reflections were merged. Data were collected and integrated using the Bruker SAINT[2] software package. The linear absorption coefficient, θ, for Mo-K radiation is 10.46 cm^{-1}. Data were corrected for absorption effects using the multi-scan technique (SADABS[3]), with minimum and maximum transmission coefficients of 0.807 and 0.939, respectively. The data were corrected for Lorentz and polarization effects. The structure was solved by direct methods[4]. The material crystallizes with one half-molecule in the asymmetric unit, residing on a two-fold rotation axis. The Ni-thiophene-Ni fragment is disordered and was modeled in two orientations with equal proportions. All non-hydrogen atoms were refined anisotropically. All hydrogen atoms were placed in calculated positions. The final cycle of full-matrix least-squares refinement[5] on F^2 was based on 4651 reflections and 284 variable parameters and converged (largest parameter shift was 0.00 times its esd) with unweighted and weighted agreement factors of:

$$R1\ (I>2.00\ (I)) = ||Fo| - |Fc|| / |Fo| = 0.033$$

$$wR2\ (\text{all data}) = [\ (\ w\ (Fo^2 - Fc^2)^2\ )/\ w(Fo^2)^2]^{1/2} = 0.075$$

The standard deviation of an observation of unit weight[6] was 1.05. The weighting scheme was based on counting statistics. The maximum and minimum peaks on the final difference Fourier map corresponded to 0.43 and –0.36 $e^-/Å^3$, respectively. Neutral atom scattering factors were taken from Cromer and Waber[7]. Anomalous dispersion effects were included in Fcalc[8]; the values for Δϕ' and Δϕ" were those of Creagh and McAuley[9]. The values for the mass attenuation coefficients are those of Creagh and Hubbell[10]. All refinements were performed using the SHELXL-2016[11] via the OLEX2[12] interface.

X-ray diffratction of Complex 5.

An orange irregular crystal of $C_{26}H_{46}NiP_2S_2$ having approximate dimensions of 0.05 x 0.11 x 0.24 mm was mounted on a cryo-loop. All measurements were made on a Bruker APEX DUO diffractometer with a TRIUMPH curved-crystal monochromator with Mo-Kα radiation. The data were collected at a temperature of -183.0 ± 0.1°C to a maximum 2θ value of 61.1°. Data were collected in a series of ϕ and ω scans in 0.5° oscillations using 10.0-second exposures. The crystal-to-detector distance was 40.15 mm. Of the 36594 reflections that were collected, 8427 were unique (R_{int} = 0.051); equivalent reflections were merged. Data were collected and integrated using the Bruker SAINT[2] software package. The linear absorption coefficient, θ, for Mo-K radiation is 9.81 cm^{-1}. Data were corrected for absorption effects using the multi-scan technique (SADABS[3]), with minimum and maximum transmission coefficients of 0.844 and 0.953, respectively. The data were corrected for Lorentz and polarization effects. The structure was solved by direct methods[4]. The material crystallizes with the bis-thiophene disorder in two orientations. The two orientations are related by a 180 degree rotation about an axis parallel to the C_4-C_5 bond. All non-hydrogen atoms were refined anisotropically. All hydrogen atoms were placed in calculated positions. The final cycle of full-matrix least-squares refinement[5] on

F^2 was based on 8427 reflections and 318 variable parameters and converged (largest parameter shift was 0.00 times its esd) with unweighted and weighted agreement factors of:

$$R1\ (I>2.00\ (I)) = ||Fo| - |Fc|| / |Fo| = 0.036$$

$$wR2\ (\text{all data}) = [\ (\ w\ (Fo^2 - Fc^2)^2\ )/\ w(Fo^2)^2]^{1/2} = 0.085$$

The standard deviation of an observation of unit weight[6] was 1.03. The weighting scheme was based on counting statistics. The maximum and minimum peaks on the final difference Fourier map corresponded to 1.59 and –0.58 $e^-/Å^3$, respectively. Neutral atom scattering factors were taken from Cromer and Waber[7]. Anomalous dispersion effects were included in Fcalc[8]; the values for f' and f" were those of Creagh and McAuley[9]. The values for the mass attenuation coefficients are those of Creagh and Hubbell[10]. All refinements were performed using the SHELXL-2016[11] via the OLEX2[12] interface.

X-ray Absorption Spectroscopy

All XAS samples except Ni(dtbpe)bithiophene complex (**5**) were analyzed as solids under anaerobic conditions and diluted in boron nitride (20-50% by weight). **5** was prepared in dry toluene solvent and treated with extra bithiophene at RT to avoid the formation of S-C insertion byproduct Ni(dtbpe)(η_2-C,S-bithiophene) and quickly frozen under liquid nitrogen environment. XAS Ni K-edges were acquired at the SSRL beamline 7-3, which is equipped with a Si(220) ϕ = 90° double crystal monochromator, a 9 keV cutoff mirror, and a He cryostat (at 20 K). Data were collected using a Canberra 30-element Ge solid-state detector with a 3mm Co filter. Data averaging and energy calibration were performed using SixPack[26],The AUTOBK algorithm available in the Athena software package[27] was employed for data reduction and normalization. $[Ni(dtbpe)]_2$-arene complex and $Ni(dtbpe)Cl_2$ were used as reference to evaluate the oxidation state.

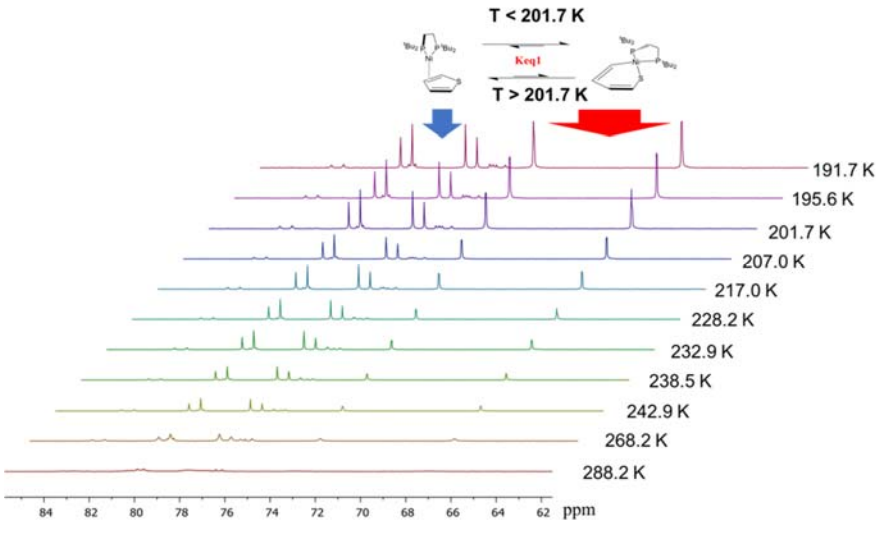

Supplementary Figure 1. ***^{31}P NMR spectra*** *(400 MHz, THF-d_8, 191.7K-288.15K) that show the effect of temperature on the equilibrium betwee the Ni(dtpe)thiophene π adduct complex and the C-S insertion product. Within the equilibrium mixture, insertion product is favoured at lower temperatures*

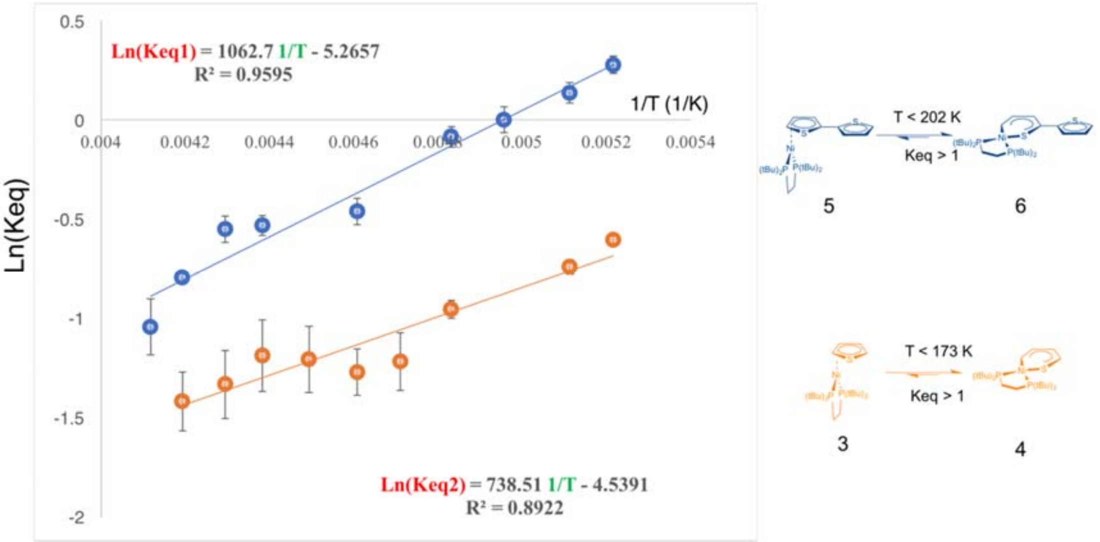

Supplementary Figure 2. *Van't Hoff plots for equilib*ria between complexes **3-4** (blue) and **4-5** (orange).

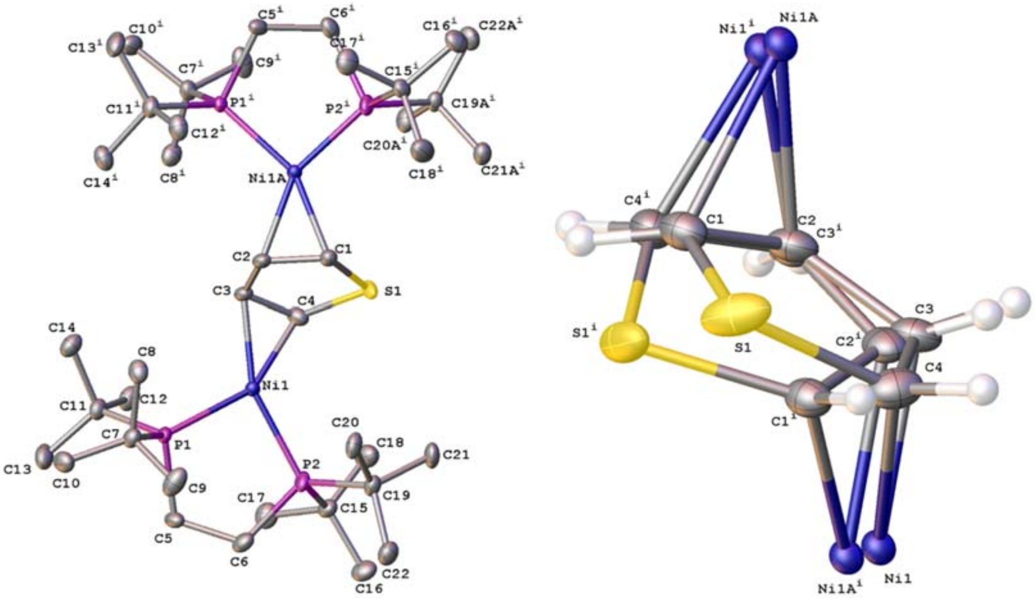

Complex 2 – CCDC #1587316

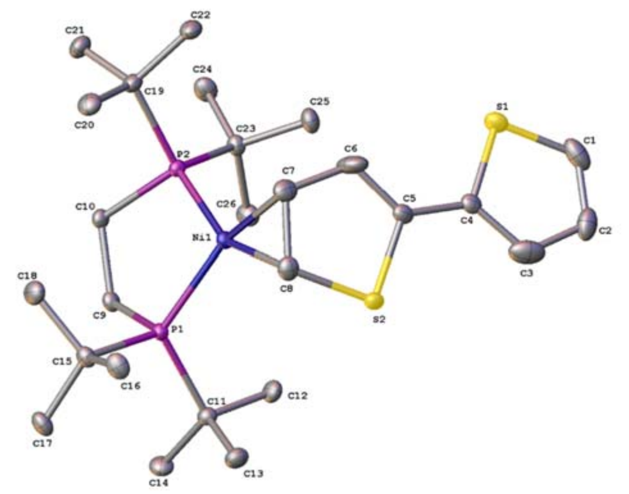

Complex 5 – CCDC #1587317

Supplementary Figure 3. *ORTEP representation of **2** and **5**, depicting thermal ellipsoids at the 50% probability level.* ***The relative*** *orientations of the two Ni-thiophene-Ni disordered fragments of **complex 2** are presented on the top right.*

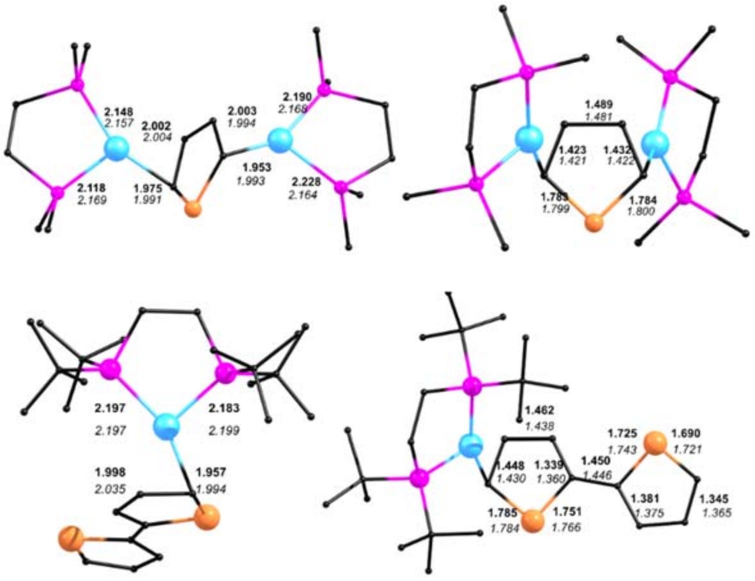

Supplementary Figure 4. *Experimental (bold) and calculated (italics) geometrical parameters for **5H**-Ni(dtbpe)bithiophene and **2H**-[Ni(dtbpe)]$_2$thiophene). Bond distances in Å, calculations were performed at the B3LYP[14]/Def2-TZVP[15] level of theory using ORCA 3.03 package[13]. Computational efficiency was improved by applying the RI approximation (RIJCOSX) for the hybrid functional[19]*

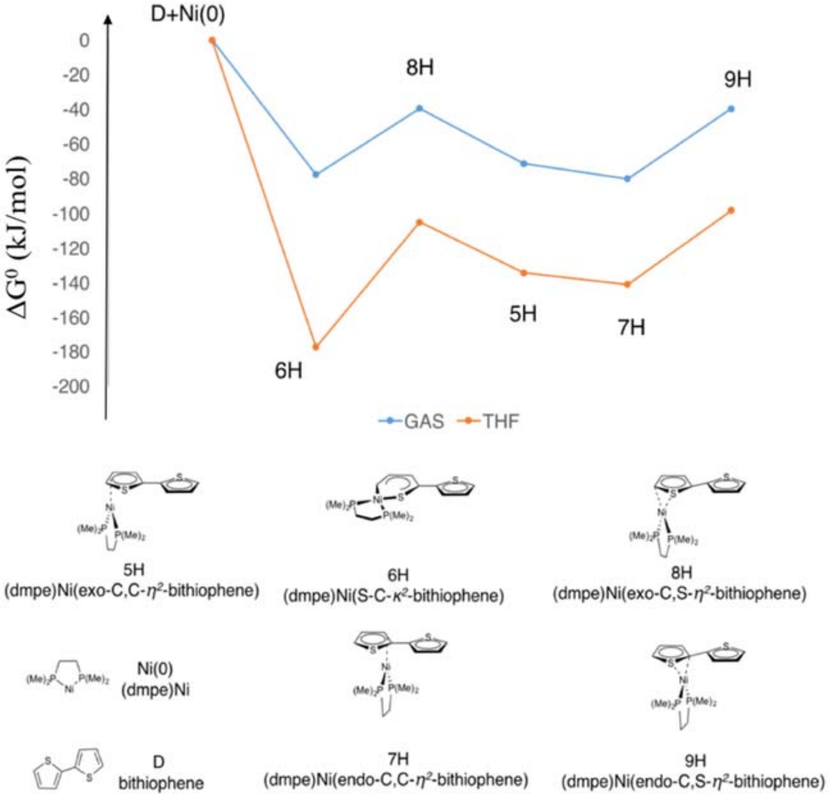

Supplementary Figure 5. ***Gibbs free energy profile of possible products.*** *Optimizations were calculated at the B3LYP/def$_2$-TZVP level of theory. Gibbs free energies were derived in gas phased and THF solvent (298.15K) at the M06/def$_2$-TZVP ZORA and M06-SMD[16]/def$_2$-TZVP ZORA level, repectively. Gibbs Free Energy of isolated Ni(dmpe) fragment (**Ni(0)**) and bithiophene (**D**) arbitrarily set to 0 kJ/mol. Detail results are listed in Table S5.*

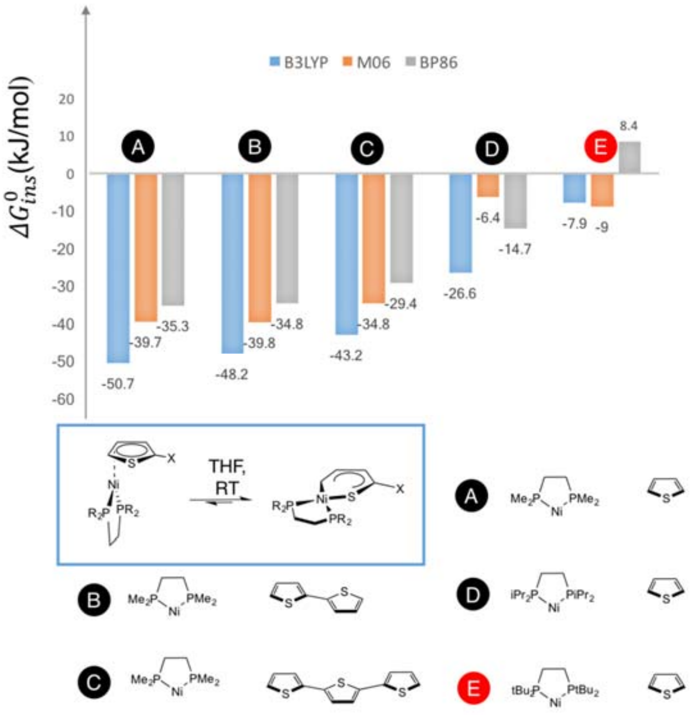

Supplementary Figure 6. ***Gibbs Energy difference between insertion product and π-adduct product ($\Delta\Delta G_{\pi\text{-}ins}$). Geometry*** *optimization was calculated at the B3LYP/def2-TZVP level of theory. Gibbs free energies were derived in at the B3LYP/def2-TZVP ZORA, M06/def2-TZVP ZORA and M06-SMD/def2-TZVP ZORA level, repectively.*

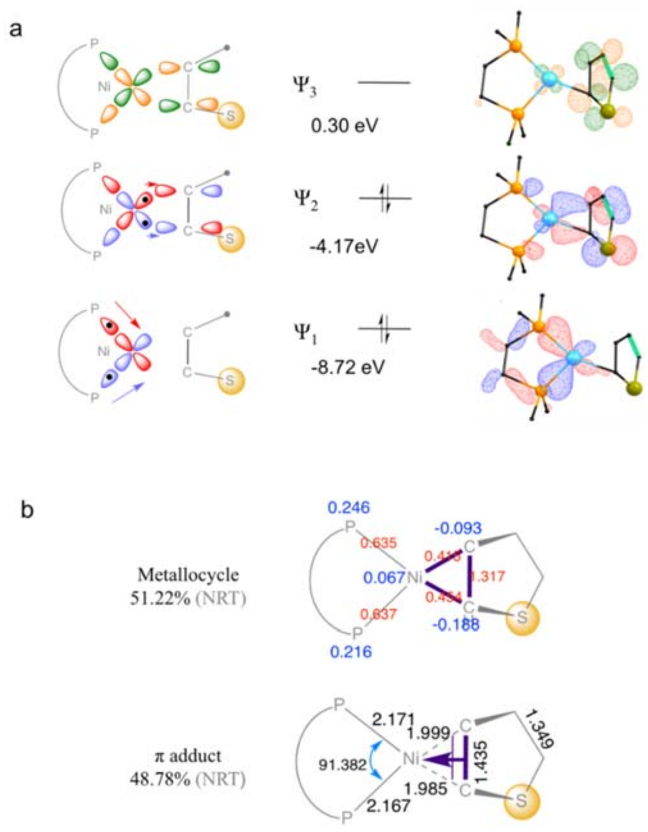

Supplementary Figure 7. ***DFT results for Ni(dmpe)thiophene***. *(a) the MO energy diagram involved in three center four electron charge transfer model of square planar Ni(phosphine)thiophene complexes. (b) NRT results and Mulliken charge distribution (blue) and Bond order Index (red), calculated geometry bond distance and angle (black). NRT (natural resonance theory) calculation shows metallocycle resonance structure is slightly more populated than π adduct resonance structure.*

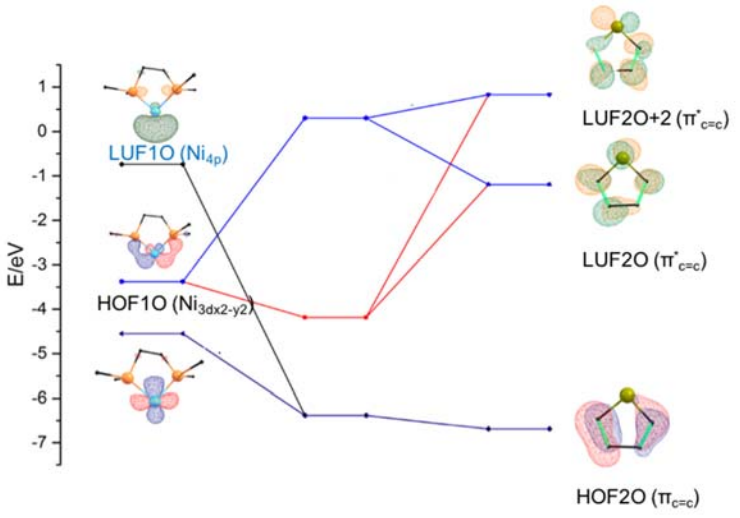

Supplementary Figure 8. *Molecular Orbital Decompsition analysis of important frontier orbitals in Ni(dmpe)thiophene complex* ***3H*** *system.*

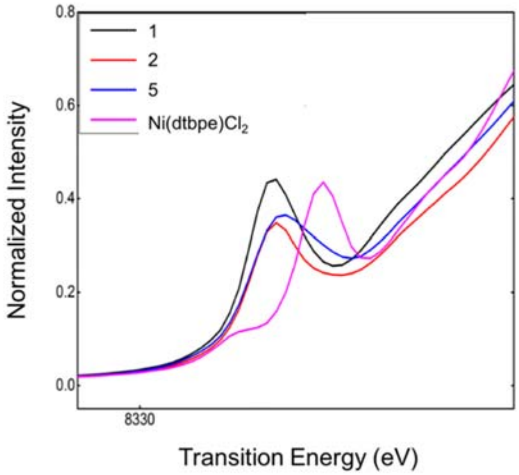

	code	1	2	5	Ni(dtbpe)Cl_2
1s→4p	transition Energy (eV)	8334.18	8334.32	8335.37	8335.84

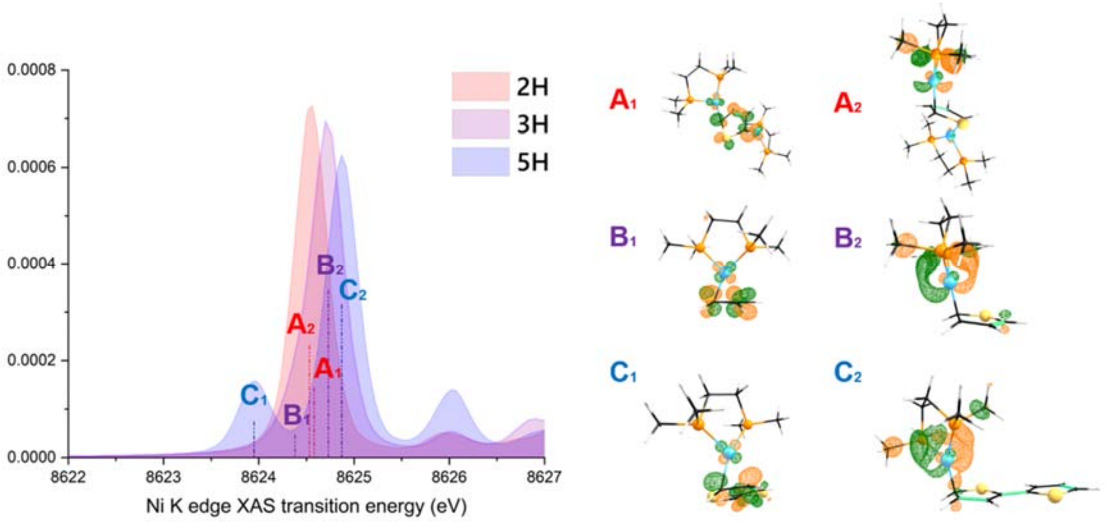

Supplementary Figure 9. ***Ni K-edge XAS data.*** *(top) experimental spectrum with 1s – 4p transition energy results abtained by fitting. (bottom) TD-DFT Ni K-edge pre-edge calculated spectra. π backbonding orbitals with Ni 3d character allows 1s-3d Ni K edge transition are presented as A_1,B_1,C_1, Ni 4p orbital allows 1s-4p Ni K edge transition are visualized as A_2, B_2, C_2. Calcuation details can be found in Table S10.*

The 1s-3d pre-edge feature reflects the degree of electron depletion in the $3d_{x2-y2}$ orbital through π backbonding. In theory, the intensity of the feature therefore reflects the degree of metal backbonding although even small mixing of 4p character has a very large influence on the final intensity of this feature. The intense pre-edge 1s-$4p_z$ transition feature correspond to a localized atomic transition where the energy of this transition directly reflets electron density at the metal centre. See more details in ref **36** in main manuscript.

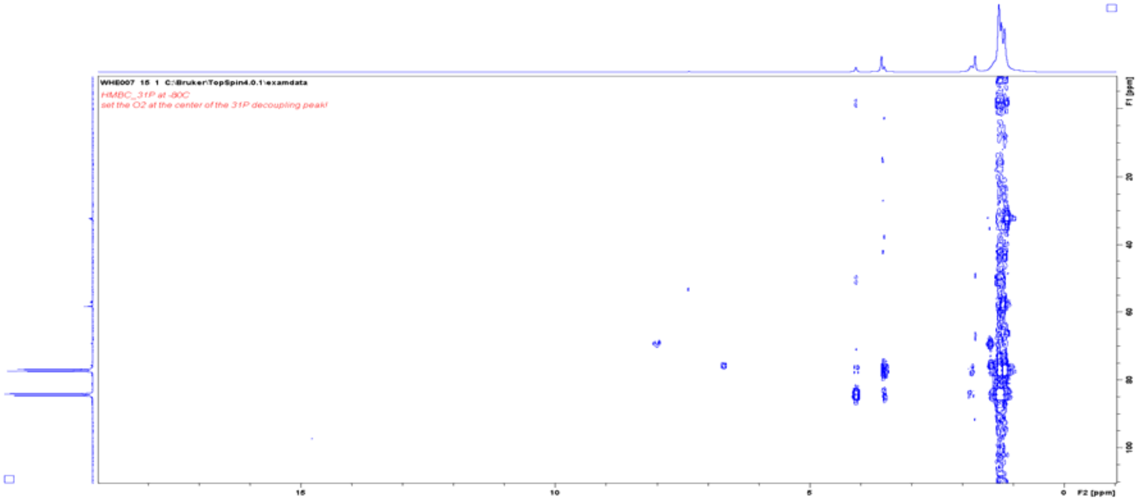

Supplementary Figure 10. *2D HSQC NMR of Complex **2**. Strong coupling between P and $H_{(TH)}$ were found.*

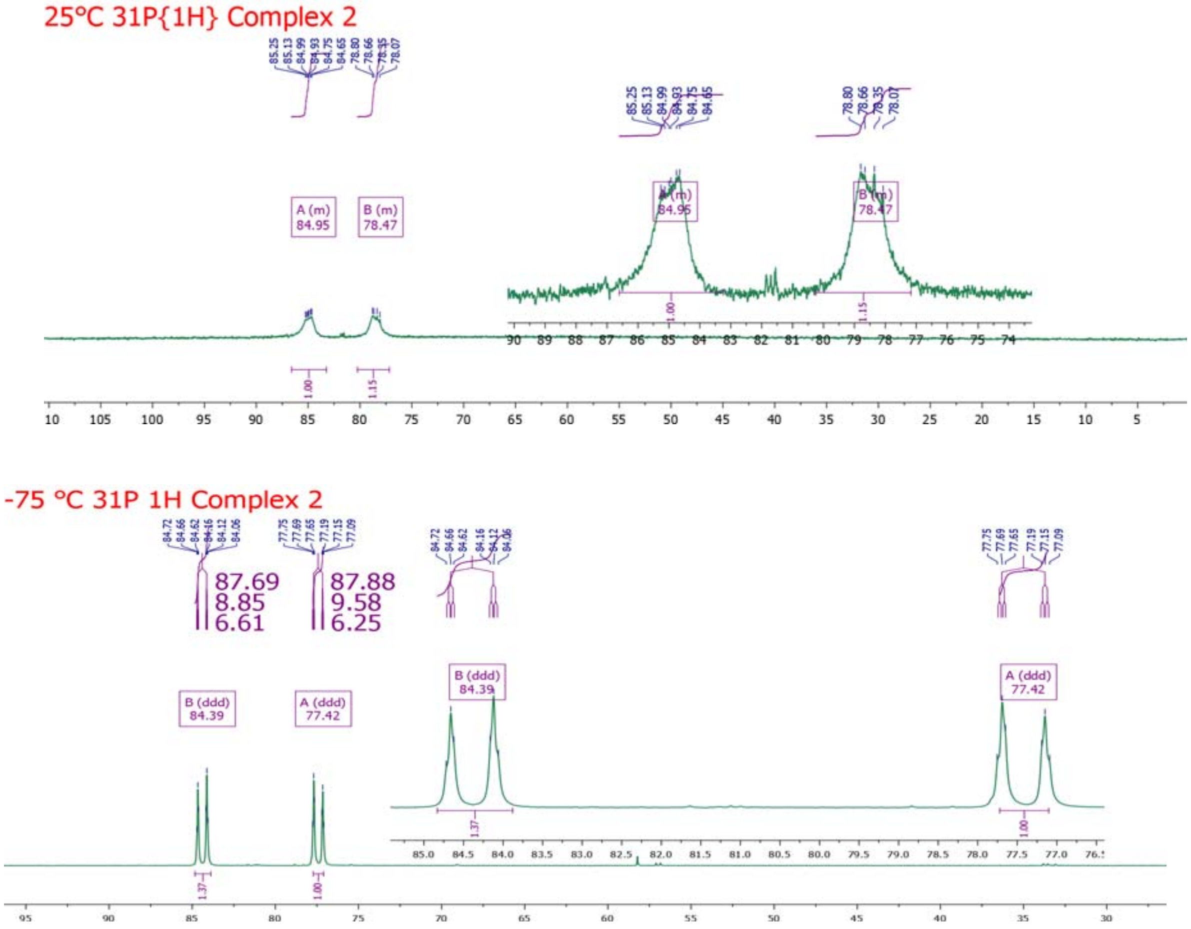

Supplementary Figure 11. *$^{31}P\{^1H\}$ NMR spectrum (162 MHz, 298K & 208K, THF-d_8) of **2**. Inset shows long distance $^5J_{P,P}$ coupling.*

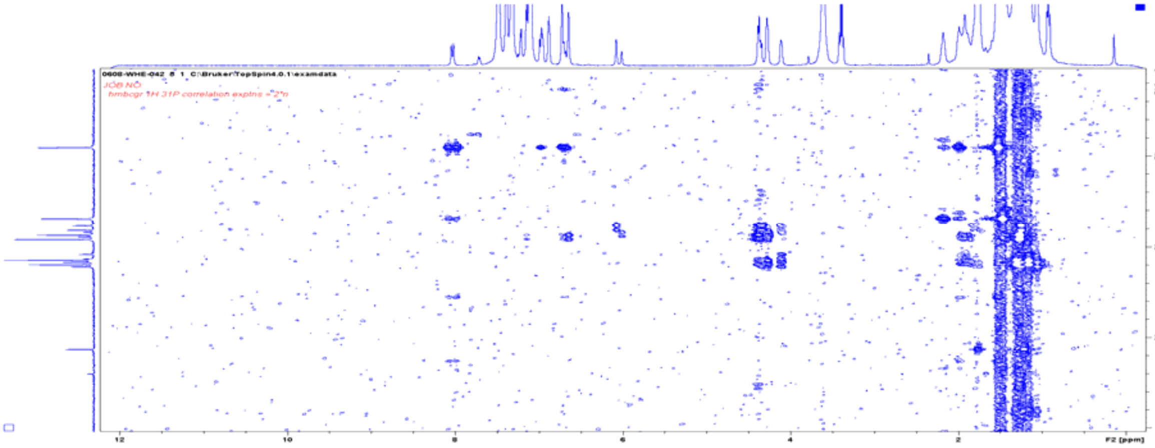

Supplementary Figure 12. *2D HSQC NMR of Complex **3 & 4**. Strong coupling between P and $H_{(TH)}$ were found in both π adduct species and insertion side product, which allows for assignment of the proton signals.*

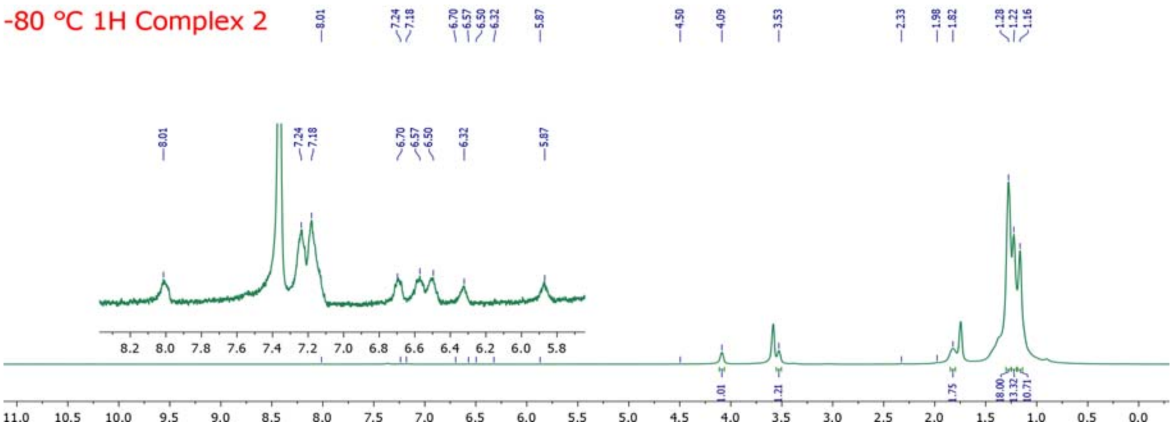

Supplementary Figure 13. *^{1}H NMR (400 MHz, 298K & 208K, THF-d_8) of **2**.*

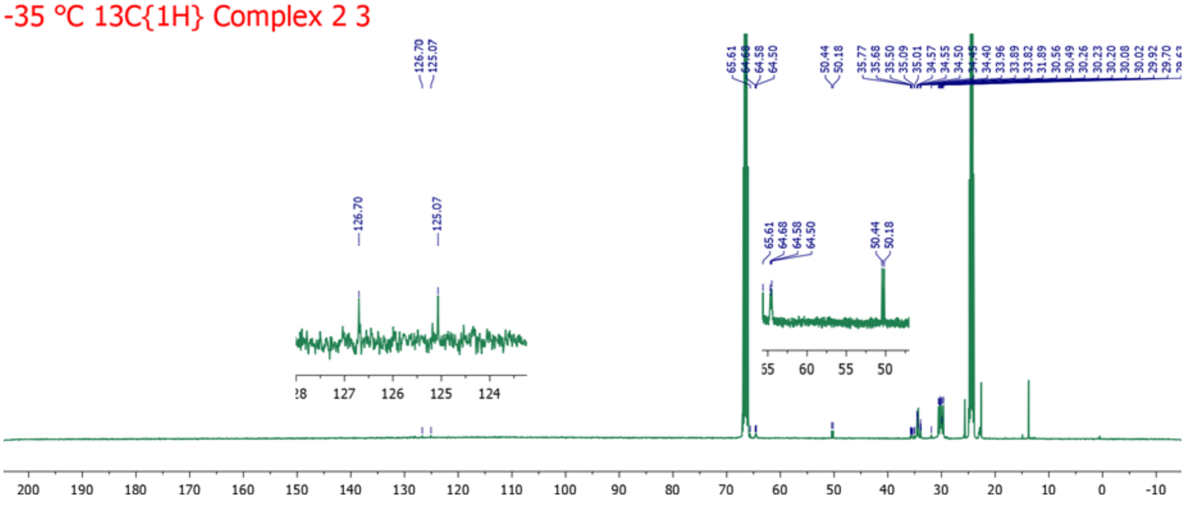

Supplementary Figure 14. *$^{13}C\{H\}$ NMR spectrum (162 MHz, 238K, THF-d_8) of **2 & 3**.*

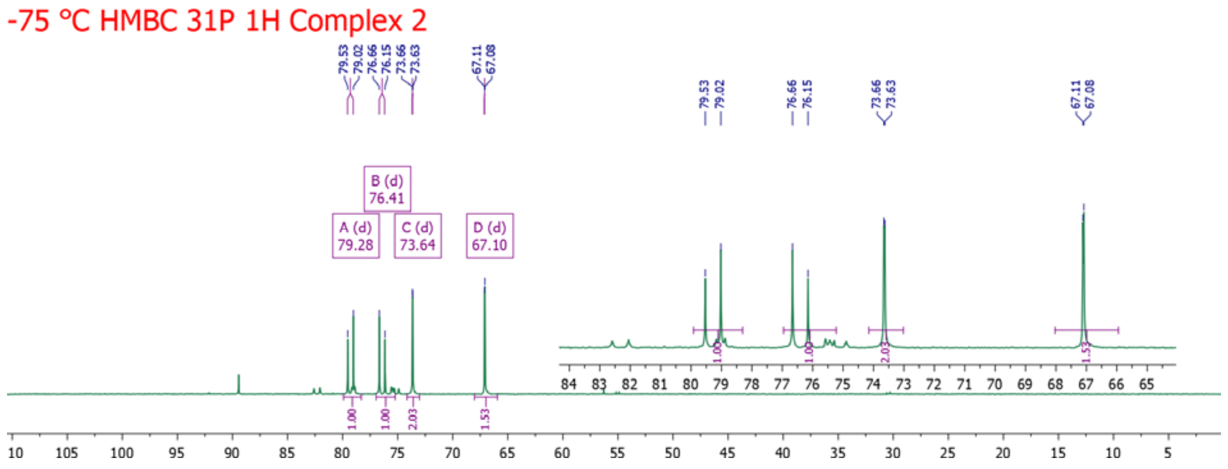

Supplementary Figure 15. $^{31}P\{^{1}H\}$ NMR spectrum (162 MHz, 198K, THF-d_8) of **3 & 4**. Inset shows distance $^{5}J_{P,P}$ coupling.

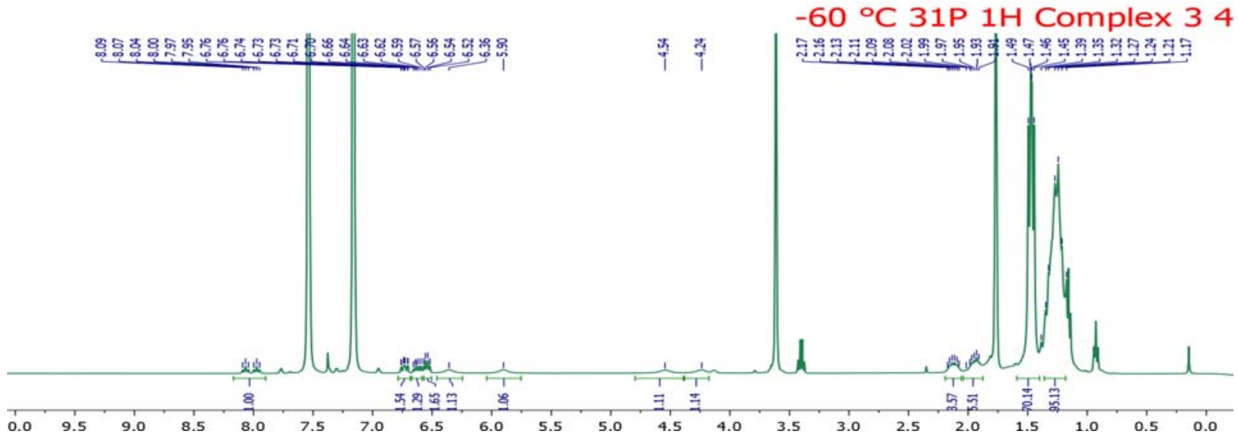

Supplementary Figure 16. *^{1}H NMR (400 MHz, 218K, THF-d_8) of* ***3 & 4**.*

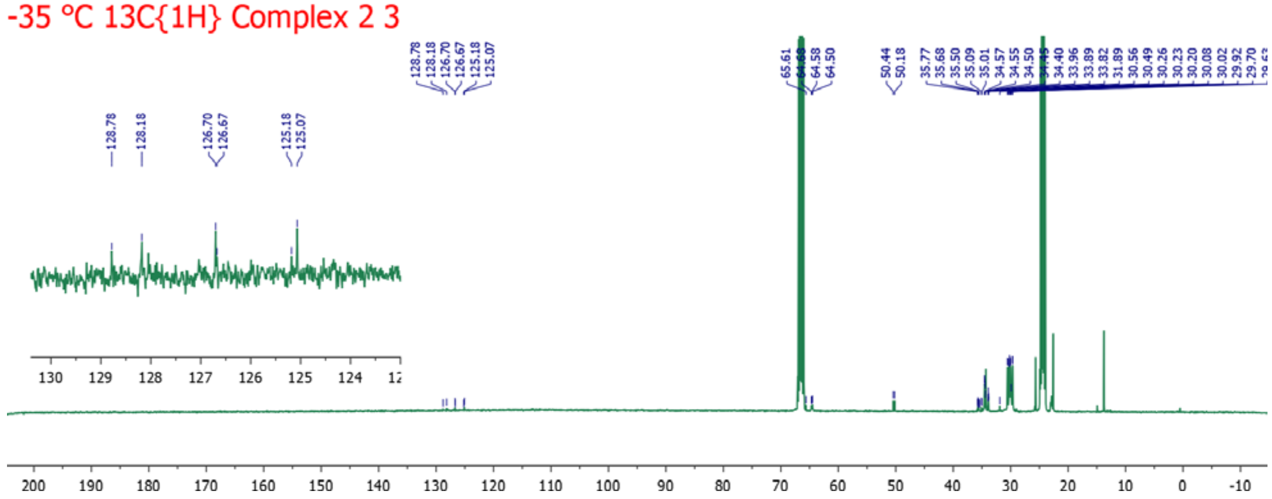

Supplementary Figure 17. *$^{13}C\{H\}$ NMR spectrum (162 MHz, 238K, THF-d_8) of **3 & 4**.*

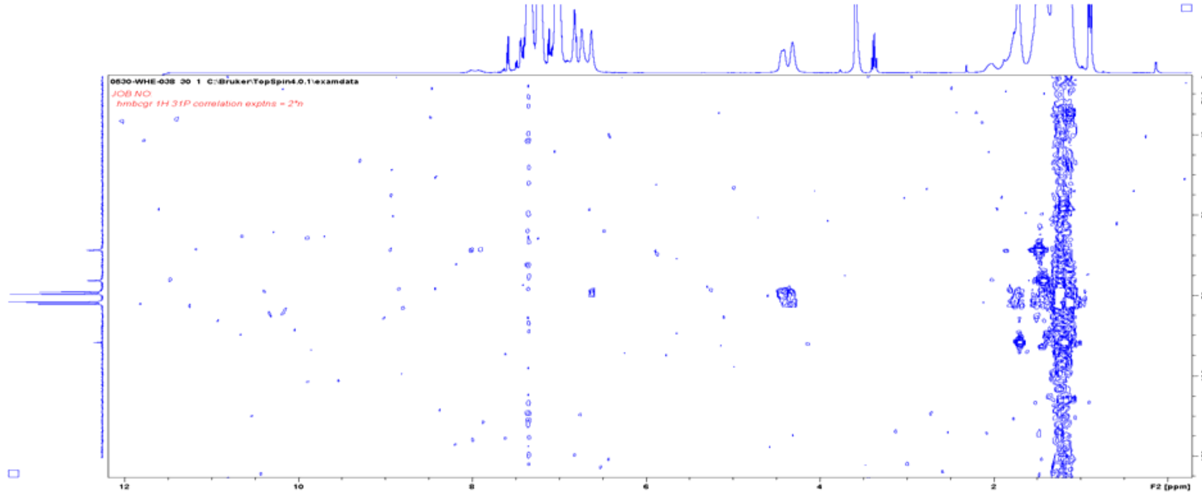

Supplementary Figure 18. *2D NMR of Complex **5 & 6**. Strong coupling between P and $H_{(TH)}$ were found.*

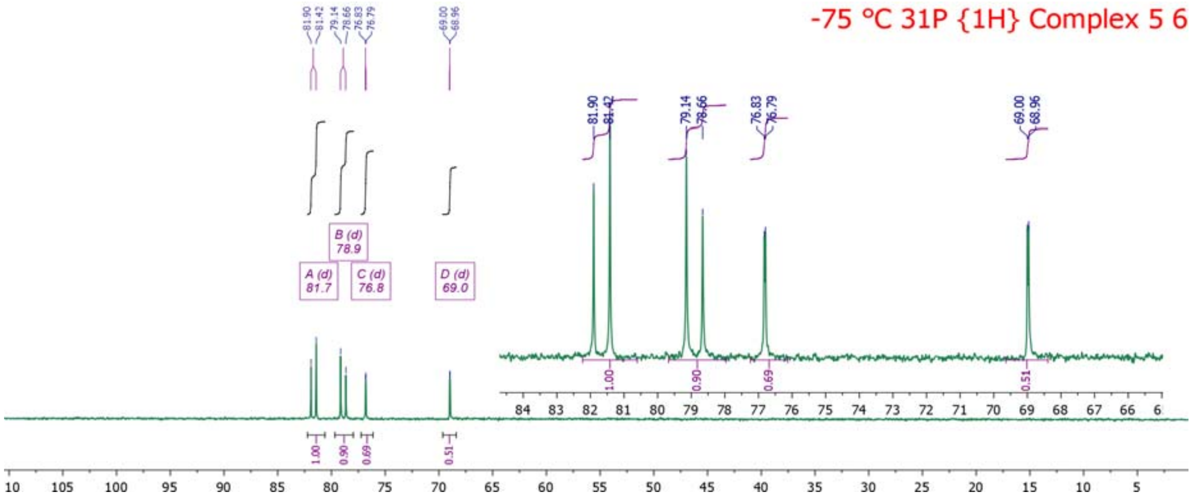

Supplementary Figure 19. *$^{31}P\{^{1}H\}$ NMR spectrum (162 MHz, 198K, THF-d_8) of* ***5 & 6****. Inset shows distance $^{5}J_{P,P}$ coupling.*

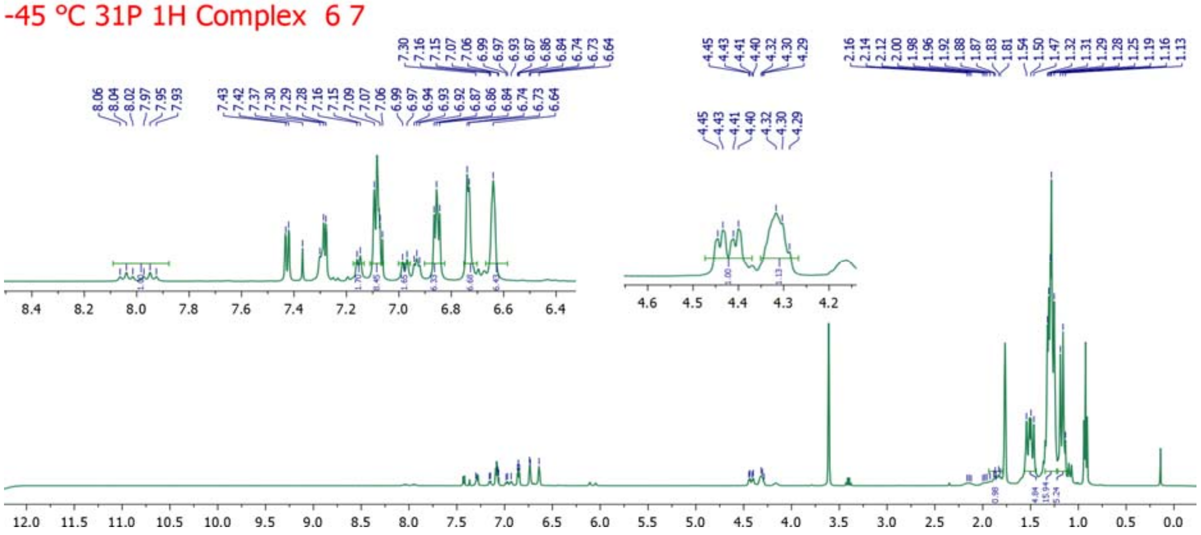

Supplementary Figure 20. *^{1}H NMR (400 MHz, 218K, THF-d_8) of* ***5 & 6.***

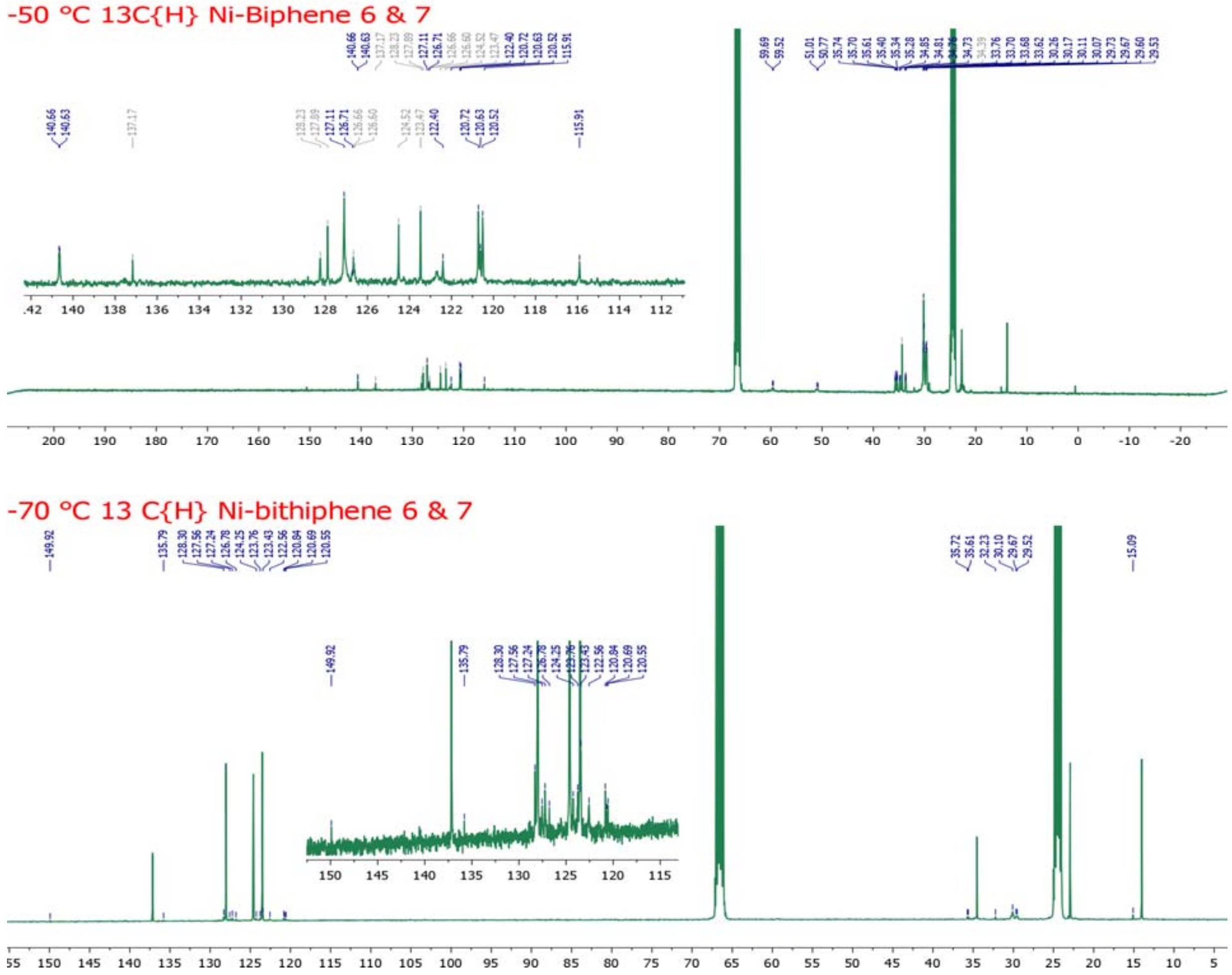

Supplementary Figure 21. *^{13}C NMR (101 MHz, 223K & 203K, THF-d_8) of* ***5*** *and* ***6****. The assignment of 6 and 7 were found by the intensity change at different temperature.*

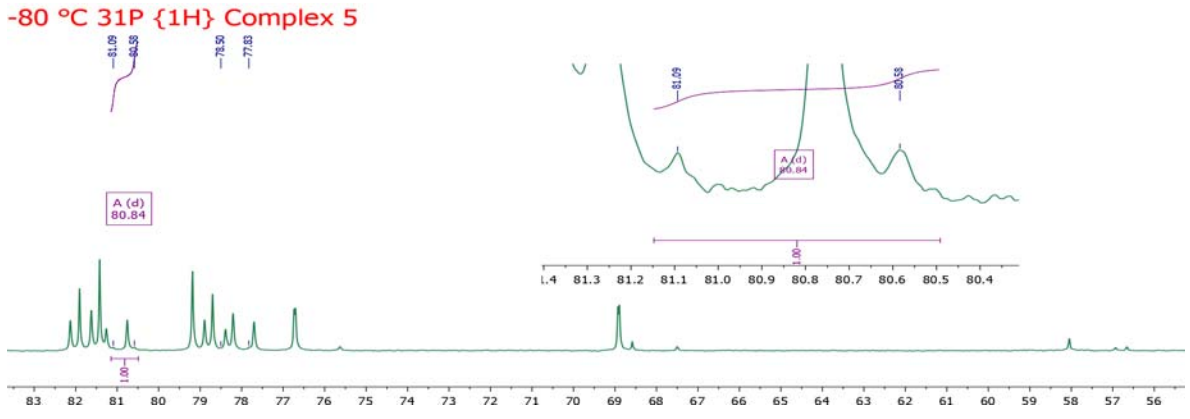

Supplementary Figure 22. *^{31}P NMR (162 MHz, THF-d_8) δ 80.84 (d, J = 82.5 Hz) of* ***7*****.** *Inset shows distance $^5J_{P,P}$ = 82.5 Hz coupling, indicative of Ni(0) complex.*

	ΔH^0_{INS}	ΔS^0_{INS}	T_{eq}
Thiophene (3=4)	-8.8 ± 0.7 kJ/mol	-44 ± 3 *J*/(*mol K*)	202 ± 5 *K*
Bithiophene (5=6)	-6.2 ± 0.8 *kJ/mol*	-38 ± *4 J*/(*mol K*)	173 ± 5 *K*

Supplementary Table 1. *Experimentally determined thermodynamic parameters for the C-S cleavage process in Ni(dtpbe)thiophene (3 → 4) and Ni(dtpbe)bithiophene (5 → 6).*

**3 → 4**				**5 → 6**			
**T(K)**	**1/T(1/K)**	**Ln(Kins)**	**STDEV.S**	**T(K)**	**1/T(1/K)**	**Ln(Kins)**	**STDEV.S**
242.86	0.0041176	-1.045344	0.141053	238.47	0.0041934	-1.420016	0.1474273
238.47	0.0041934	-0.796343	0.0227102	232.85	0.0042946	-1.333583	0.1702991
232.85	0.0042946	-0.552353	0.0672726	228.16	0.0043829	-1.189128	0.1809951
228.16	0.0043829	-0.531983	0.0502451	222.53	0.0044938	-1.208677	0.1672291
217.01	0.0046081	-0.46294	0.066883	217.01	0.0046081	-1.27286	0.1174897
206.96	0.0048319	-0.085911	0.0518812	212.29	0.0047105	-1.220385	0.1454121
201.74	0.0049569	0.0002931	0.0660025	206.96	0.0048319	-0.956522	0.0453005
195.59	0.0051127	0.135611	0.051926	195.59	0.0051127	-0.742808	0.0362195
191.7	0.0052165	0.2775597	0.0430438	191.7	0.0052165	-0.605227	0.0267185

Supplementary Table 2. *VT NMR experiment temperature (T), reciprocal temperature (1/T), average of insertion process from π adduct to S-C insertion product equilibrium constant (K_{ins}) calculated using the ^{31}P {^{1}H} peaks intensity.*

Compound Number	2 [Ni(dtpe)]$_2$thiophene	5 Ni(dtpe)bithiophene
**Empirical Formula**	$C_{40}H_{84}Ni_2P_4S$	$C_{26}H_{46}NiP_2S_2$
**Formula Weight**	838.43	543.40
**Crystal Colour, Habit**	orange, irregular	orange, irregular
**Crystal Dimensions**	0.06 x 0.10 x 0.13 mm	0.05 x 0.11 x 0.24 mm
**Crystal System**	monoclinic	monoclinic
**Space Group**	C 2/c (#15)	P 21/n (#14)
**Lattice Type**	C-centered	Primitive
***a*, Å**	26.060(2)	9.2022(11)
***b*, Å**	11.4151(9)	17.143(2)
**c, Å**	15.3575(11)	17.634(2)
***α*, °**	90	90
**β, °**	98.329(2)	96.401(3)
**γ, °**	90	90
**V, Å^3**	4520.4(6)	2764.5(6)
**Z value**	4	4
**Dcalc, g/cm^3**	1.23	1.31
**F_{000}**	1824.00	1168.00
**μ(Mo-Kα), cm^{-1}**	10.46	9.81
**Data Images (no., t/s)**	1964, 20	1078, 10
**2θ max, °**	52.80	61.10
**Reflections measrd**	39334	36594
**Unique reflcn, R_{int}**	4651, 0.062	8427, 0.051
**Absorption, T_{min}, T_{max}**	0.807, 0.939	0.844, 0.953
**Observed data (I>2.00 σ(I))**	3604	6700
**No. parameters**	284	318
**R1, wR2 (F^2, all data)**	0.054, 0.075	0.054, 0.085
**R1, wR2 (F, I>2.00 σ(I))**	0.033; 0.067	0.036, 0.079
**Goodness of Fit**	1.05	1.03
**Max, Min peak, e–/Å^3**	0.43, -036	1.59, -0.58

Supplementary Table 3. *Crystallographic data for complex **2** and **5**.*

5H	P1-Ni1	P2-Ni1	Ni-C7	Ni-C8	C7-C8	S2-C8	S2-C5
XRD	2.197	2.183	1.998	1.957	1.447	1.785	1.751
Level A	2.176	2.172	2.037	1.971	1.429	1.791	1.77
Level B	2.197	2.199	2.035	1.994	1.43	1.784	1.766
σ_A	0.96%	0.50%	1.91%	0.74%	1.21%	0.33%	1.05%
σ_B	0.01%	0.75%	1.80%	1.86%	1.20%	0.03%	0.85%
δ_A	0.021	0.011	-0.039	-0.014	0.018	-0.006	-0.019
δ_B	0.000	-0.016	-0.037	-0.037	0.017	0.001	-0.015
**5H**	**C5-C6**	**C6-C7**	**C1-C2**	**C2-C3**	**C3-C4**	**C1-S1**	**C4-S1**
XRD	1.339	1.462	1.345	1.405	1.381	1.69	1.725
Level A	1.359	1.435	1.363	1.419	1.375	1.724	1.746
Level B	1.36	1.438	1.365	1.419	1.375	1.721	1.743
σ_A	1.53%	1.85%	1.28%	0.99%	0.41%	1.97%	1.17%
σ_B	1.57%	1.65%	1.45%	0.97%	0.44%	1.79%	1.04%
δ_A	-0.020	0.027	-0.018	-0.014	0.006	-0.034	-0.021
δ_B	-0.021	0.024	-0.020	-0.014	0.006	-0.031	-0.018

2H	P1-Ni1	P2-Ni1	Ni1-C3	Ni-C4	C3-C4	C1-C2
XRD	2.19	2.228	2.003	1.953	1.432	1.423
Level A	2.168	2.164	1.994	1.993	1.422	1.421
Level B	2.18	2.182	2.017	2.013	1.417	1.417
σA	1.03%	2.91%	0.46%	2.04%	0.63%	0.18%
σB	0.48%	2.09%	0.69%	3.04%	1.04%	0.42%
δA	0.022	0.064	0.009	-0.040	0.010	0.002
δB	0.010	0.046	-0.014	-0.060	0.015	0.006

Supplementary Table 4. *Comparison of selected bond distances for **5** and **2**, and their corresponding simplified DFT-calculated distances for **5H** and **2H**. Bond distances are listed in Å, calculations of geometry optimizations were carried out in the gas phase using ORCA 3.03 program at B3LYP/ def_2[18]-TZVP level. Available experimental crystal structures were compared to results from two theoretical levels: A = B3LYP/def_2-TZVP ZORA[20] (Grid6) and B = B3LYP/def_2-TZVP(Grid4). σ_A and σ_B are % deviation from XRD. δ_A and δ_B are standard deviations relative to XRD. (Root mean square deviation **(RMSD)** value: δA_{5H} 0.079, δB_{5H} 0.080, δA_{2H} 0.080 δB_{2H} 0.079).*

Code	M06[17], GAS (kJ/mol)		M06-SMD(THF) (kJ/mol)	
	ΔH^0_{ins}	ΔG^0_{ins}	ΔH^0_{ins}	ΔG^0_{ins}
**D+Ni0**	0.0	0.0	0.00	0.0
**6H**	-144.0	-78.2	-175.6	-177.7
**8H**	-101.9	-39.9	-107.3	-105.3
**5H**	-135.8	-71.9	-135.7	-134.7
**7H**	-142.7	-80.7	-143.5	-141.5
**9H**	-101.0	-40.0	-101.2	-98.6

Supplementary Table 5. ***Entropy and Gibbs Free Energy results of DFT calculation of Ni(dmpe)bithiophene system.*** *Gibbs free energy of isolated Ni(dmpe) fragment and bithiophene arbitrarily set to 0 kJ/mol. Related complex structures can be found in Figure S5. (SMD THF Solevnt parameters, dielectric constant ε 7.43, Refractive index 1.407, Surface Tension 26.59 mN/m, Abraham's hydrogen bond basicity parameter 0.48, Abraham's hydrogen bond basicity parameter 0.00).*

Code	B3LYP-SMD (THF) (kJ/mol)		M06-SMD (THF) (kJ/mol)		BP86[18]-SMD (THF) (kJ/mol)	
	ΔH^0_{ins}	ΔG^0_{ins}	ΔH^0_{ins}	ΔG^0_{ins}	ΔH^0_{ins}	ΔG^0_{ins}
**6**	0.0	0.0	0.00	0.0	0.0	0.0
**5**	16.7	22.1	-6.0	-0.7	16.7	5.2
**7**	36.4	35.7	0.3	-0.4	36.4	23.9

Supplementary Table 6. ***Entropy and Gibbs Free Energy results of DFT calculation of Ni(dtbpe)bithiophene system.*** *Geometry optimization was calculated at the B3LYP/def2-TZVP level of theory. Gibbs free energies were derived in at the B3LYP/def2-TZVP, M06[17]/def$_2$-TZVP ZORA and M06-SMD/def$_2$-TZVP ZORA level, repectively. Gibbs Free Energy of C-S insertion speciese **6**, arbitrarily set to 0 kJ/mol.*

**Code**	**ligand**		**B3LYP-SMD**		**M06-SMD**		**BP86-SMD**	
	R	X	ΔH^0_{ins}	ΔG^0_{ins}	ΔH^0_{ins}	ΔG^0_{ins}	ΔH^0_{ins}	ΔG^0_{ins}
**A**	Me	H	-58.9	-50.7	-35.2	-39.7	-39.8	-35.3
**B**	Me	TH	-53.4	-48.2	-37.9	-39.8	-36.7	-34.8
**C**	Me	BiTH	-50.8	-43.2	-28.3	-34.8	-36.0	-29.4
**D**	iPr	H	-31.4	-26.6	-4.8	-6.4	-13.1	-14.7
**E**	tBu	H	-19.5	-7.9	-14.6	-9.0	2.7	8.4

Supplementary Table 7. ***For structures of these ligands, see SI appendix****. Zero-point energies, thermal corrections, and entropies are calculated at the B3LYP/B3LYP level. Energies are in kJ/mol. Gibbs free energies and Enthalpy in the energy profile are calculated at 298 K at the B3LYP//M06//BP86-SMD. Combination of thiophene(TH), bithiophene(TH-TH), terthiophene(TH-BiTH) and dmpe, dippe, dtbpe phosphine ligands are used to evaluate the steric effects.*

Code	Bonding	MO		Ni(dmpe)			thiophenic ligand		
		Orb.	E(eV)	Orb.	E (eV)	Cont.	Orb.	E (eV)	Cont.
3H	π	HOMO	-4.19	HOF1O	-3.38	51.1%	LUF2O	-1.2	22.2%
							LUF2O+2	0.83	2.4%
	π^*	LUMO+1	0.3	HOF1O	-3.38	14.9%	LUF2O	-1.2	54.3%
							LUF2O+2	0.83	4.7%
	n	HOMO-5	-6.38	LUF1O	-0.74	3.1%	HOF2O	-6.69	58.8%
5H	π	HOMO	4.19	HOF1O	-3.41	50.3%	LUF2O	-1.84	14.2%
							LUF2O+1	-0.43	7.0%
	π^*	LUMO	-0.94	HOF1O	-3.41	6.9%	LUF2O	-1.84	76.7%
							LUF2O+1	-0.43	13.4%
	π^*	LUMO+3	0.55	HOF1O	-3.41	11.7%	LUF2O	-1.84	3.3%
							LUF2O+1	-0.43	58.1%
	n	N/A	N/A	LUF1O	-0.76	<1.00%	HOF2O	-5.85	<1.00%
2H	π	HOMO-1	-3.99	HOF1O	-3.42	31.4%	LUF2O	-0.5	7.6%
							LUF2O+1	-0.19	5.2%
	π	HOMO	-3.72	HOF1O	-3.42	15.9%	LUF2O	-0.5	4.3%
							LUF2O+1	-0.19	2.6%
	π^*	LUMO+5	0.89	HOF1O	-3.42	12.7%	LUF2O	-0.5	17.4%
							LUF2O+1	-0.19	34.0%
	n	N/A	N/A	LUF1O	-0.75	<1.00%	HOF2O	-4.28	<1.00%

Ni(dmpe)→thiophenic ligand Charge transfer (CDA)			
**Code**	π_{CT}	Total CT	% π_{CT}
3H	0.14	0.15	93.9
5H	0.12	0.15	75.1
2H	0.08	0.13	92.3

Supplementary Table 8. *Molecular Orbital Decomposition Analysis (top) and Charge Decomposition Analysis (CDA) (bottom) results of important frontier fragment orbitals (**FO**) in Ni(dmpe)thiophene complex systems.*

π_{CT} is electron donation from Ni(dmpe) to the π ligand that occurs specifically through π backbonding involving the Ni $3d_{x2-y2}$ orbital. Total CT includes contributions from all MO interactions. The MO analysis indicates that there is strong π backbonding but very little (if any) σ donation from thiophene π_b electrons to the nickel centre. This is supported by the observation that the 3c-4e π interaction described in the manuscript contributes a very large fraction of the total charge redistribution between the Ni(dmpe) and thiophene fragments (see % π_{CT}).

Ni(dmpe)BT **3H**	Atom	Mulliken Charge	NPA Charge
1	Ni	0.056	-0.309
2	P	0.211	1.037
3	P	0.238	1.040
4	C	-0.363	-0.914
5	H	0.111	0.229
6	H	0.129	0.239
7	H	0.095	0.214
8	C	-0.357	-0.918
9	H	0.114	0.234
10	H	0.096	0.215
11	H	0.105	0.228
12	C	-0.357	-0.916
13	H	0.107	0.229
14	H	0.110	0.231
15	H	0.095	0.214
16	C	-0.359	-0.913
17	H	0.111	0.230
18	H	0.096	0.213
19	H	0.111	0.229
20	C	-0.218	-0.694
21	C	-0.218	-0.693
22	H	0.090	0.216
23	H	0.098	0.218
24	H	0.098	0.218
25	H	0.092	0.217
dmpe fragment		0.237	0.605
26	S	-0.102	0.335
27	C	-0.084	-0.477
28	C	-0.171	-0.349
29	C	-0.205	-0.199
30	C	0.067	-0.234
31	C	0.004	-0.216
32	S	0.004	0.421
33	C	-0.162	-0.424
34	C	-0.150	-0.246
35	C	-0.179	-0.256
36	H	0.112	0.229
37	H	0.109	0.235
38	H	0.103	0.214
39	H	0.120	0.229
40	H	0.122	0.218
41	H	0.120	0.223
bithiophene fragment		-0.293	-0.295

Ni(dmpe)TH **5H**	Atom	Mulliken Charge	NPA Charge
2	Ni	0.067	-0.296
1	P	0.246	1.042
3	P	0.216	1.052
4	C	-0.356	-0.918
5	C	-0.360	-0.916
6	C	-0.220	-0.691
7	C	-0.218	-0.696
8	C	-0.365	-0.915
9	C	-0.347	-0.918
10	H	0.099	0.218
11	H	0.089	0.215
12	H	0.096	0.216
13	H	0.093	0.218
14	H	0.108	0.231
15	H	0.097	0.214
16	H	0.113	0.232
17	H	0.109	0.227
18	H	0.096	0.214
19	H	0.112	0.231
20	H	0.097	0.214
21	H	0.103	0.226
22	H	0.114	0.232
23	H	0.094	0.212
24	H	0.118	0.240
25	H	0.111	0.231
dmpe fragment		0.244	0.611
26	S	-0.079	0.309
27	C	-0.108	-0.412
28	C	-0.251	-0.232
29	H	0.103	0.221
30	H	0.097	0.211
31	H	0.102	0.228
32	H	0.105	0.231
33	C	-0.093	-0.374
34	C	-0.188	-0.498
thiophene Fragment		-0.312	-0.316

Supplementary Table 9. *NBO charge distribution calculation results in Mulliken charge and Natural Population Analysis (NPA) chagre. NBO[21] calculations were calculated with Gaussian 09 program package[22]*

Code	Transitions	Trans. E (eV)	intensity	acceptor orbital	contr.	ΔE_{4p-3d} (eV)
5H	1s-3d	8623.95	7.38E-05	98	45.6%	0.92
				99	19.4%	
				101	18.7%	
	1s-4p	8624.87	3.15E-04	99	76.5%	
				98	15.3%	
3H	1s-3d	8624.73	3.45E-04	77	91.2%	0.35
	1s-4p	8624.38	4.70E-05	78	45.6%	
				79	22.1%	
2H	1s-3d	8624.53	2.31E-04	132	54.3%	-0.05
				133	13.0%	
				137	11.5%	
	1s-4p	8624.58	1.44E-04	132	35.4%	
				137	26.3%	
				138	14.4%	

		Nickel				Phosphine (P atoms)				Thiophene (C atoms)		
		Ni	Character			P	Character			C	Character	
Code		Total (%)	s	p	d	Total (%)	s	p	d	Total (%)	s	p
5H	98	6.5	0.4	0.8	5.3	2.2	0.4	1.2	0.6	3.7	0.2	3.5
	99	36.8	0.1	31.5	5.2	25.3	0.4	9.3	15.6	4.4	0.2	4.2
	101	12.5	0	4	8.5	3.9	0.2	1.5	2.2	15.5	1.1	14.4
	99	36.8	0.1	31.5	5.2	25.3	0.4	9.3	15.6	4.4	0.2	4.2
	98	6.5	0.4	0.8	5.3	2.2	0.4	1.2	0.6	3.7	0.2	3.5
3H	77	39.8	0.3	35.5	4	25.1	0.1	9.2	15.8	1.5	0.1	1.4
	78	10.8	1.2	0.7	8.9	3.7	1	1.4	1.3	10.1	0.6	9.5
	79	9.7	3.5	1.1	5.1	4.4	0.9	1.6	1.9	7.2	0.6	6.6
2H	132	36.7	0	32.1	4.6	24.3	0	9.3	15	1	0	1
	133	3.9	0.1	2.6	1.2	2.8	0.1	1.2	1.5	0.7	0.2	0.5
	137	10.3	0	2.6	7.7	2.6	0.6	0.9	1.1	12.1	1.4	10.7
	132	36.7	0	32.1	4.6	24.3	0	9.3	15	1	0	1
	137	10.3	0	2.6	7.7	2.6	0.6	0.9	1.1	12.1	1.4	10.7
	138	9.9	0.3	4.1	5.5	10	1.2	4.2	4.6	5.7	0.3	5.4

Supplementary Table 10. ***Ni K-edge TD-DFT results at B3LYP/dev2-TZVP level.*** *Important pre-edge transitions with details of transition energy, intensity, acceptor molecular orbital and the orbital contributions (top). MO contributions for important valuence orbitals are also listed (bottom).*

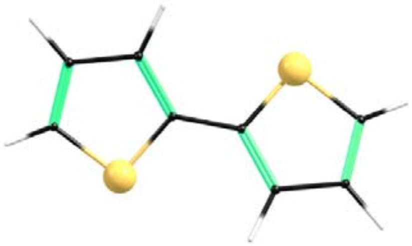

Geometry	Bithiophene (D)
E(Hatree)	-1104.70752
Gibbs(Hatree)	-1104.62748
Enthalpy(Hatree)	-1104.58449
Entropy Correction(Hatree)	-0.04299023
# Imaginary Frequencies	0

CARTESIAN COORDINATES (ANGSTROEM)

RKS B3LYP def2-TZVP def2-TZVP/C RIJCOSX Grid4

C	-1.416523	-1.91445	12.811383	H	-0.915487	-3.162002	10.845599
S	-0.553626	-1.401675	14.237667	C	-3.697314	-1.031874	13.48168
C	0.898667	-2.178585	13.74	C	-4.989878	-0.828168	12.941148
C	0.759648	-2.794149	12.532325	C	-5.0875	-1.244683	11.644958
C	-0.549437	-2.632054	12.026973	S	-3.607872	-1.896522	11.070116
C	-2.811649	-1.598142	12.592704	H	-3.426796	-0.771149	14.495787
H	1.772745	-2.13476	14.368459	H	-5.811785	-0.394976	13.492956
H	1.534619	-3.339959	12.015127	H	-5.947678	-1.2122	10.995728

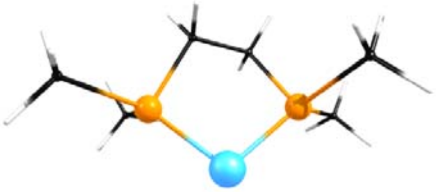

Geometry	Ni(dmpe) (Ni0)
E(Hatree)	-2462.0576
Gibbs(Hatree)	-2461.8876
Enthalpy(Hatree)	-2461.8341
Entropy Correction(Hatree)	-0.053460
# Imaginary Frequencies	0

CARTESIAN COORDINATES (ANGSTROEM)

RKS B3LYP def2-TZVP def2-TZVP/C RIJCOSX Grid4

P	-1.235059	1.792756	11.453607	H	-2.205657	2.700026	13.504645
Ni	-0.219224	-0.031313	11.446161	H	-1.730831	4.025066	12.418867
P	1.235481	0.227708	9.975578	H	-0.503778	3.1379	13.355205
C	-1.441636	3.043144	12.805798	H	-2.873961	1.408879	9.672442
C	-2.860353	2.004763	10.584994	H	-3.05892	3.050314	10.331636
C	-0.065664	2.734141	10.319592	H	-3.657957	1.638941	11.233783
C	0.564324	1.825384	9.248903	H	0.61495	-1.005723	7.964592
C	1.56502	-0.711684	8.412767	H	2.12055	-1.621011	8.647544
C	2.965984	0.730915	10.420597	H	2.13598	-0.126256	7.686759
H	0.716333	3.146207	10.964134	H	3.496473	1.166679	9.569431
H	-0.574152	3.587109	9.857153	H	3.517285	-0.139965	10.778527
H	-0.193876	1.529588	8.518687	H	2.939782	1.460445	11.230787
H	1.344371	2.359332	8.694998				

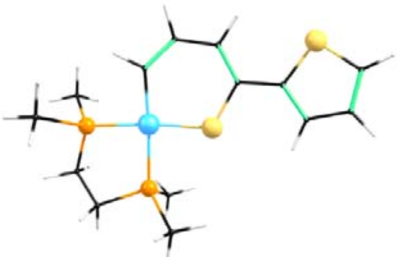

Geometry	6H
E(Hatree)	-3543.77949
Gibbs(Hatree)	-3543.50346
Enthalpy(Hatree)	-3543.43216
Entropy Correction(Hatree)	-0.0712987
# Imaginary Frequencies	0

CARTESIAN COORDINATES (ANGSTROEM)

RKS B3LYP def2-TZVP def2-TZVP/C RIJCOSX Grid4

C	0.075299	0.0671	0.085509	H	-1.415531	2.003628	1.610558
P	0.044636	0.11053	1.914988	H	0.184927	2.579467	2.052602
C	1.835254	0.1926	2.318092	H	-1.600534	2.821749	3.940412
Ni	-1.239561	-1.348404	3.122056	H	-0.162419	1.93009	4.41066
C	-2.39735	-2.442777	4.163698	H	-2.210563	1.322107	6.456966
C	-2.515473	-3.794131	4.201354	H	-0.903147	0.111425	6.33041
C	-1.817523	-4.787613	3.451588	H	-2.581885	-0.412288	6.405075
C	-0.864918	-4.577663	2.499028	H	-4.412686	0.060972	4.149455
C	-0.25783	-5.687119	1.75748	H	-3.911351	1.158882	2.862292
C	-0.572308	1.825873	2.28356	H	-4.030687	1.754785	4.534771
C	-1.029938	1.910379	3.74522	H	-2.103374	-5.814572	3.658861
P	-2.014265	0.397069	4.19462	H	-3.255133	-4.204988	4.889537
C	-1.918607	0.362867	6.023763	H	-3.091658	-1.953826	4.849431
S	-0.295528	-2.992271	2.058119	C	0.687608	-5.640828	0.759233
C	-3.759305	0.900479	3.910962	C	1.044	-6.911409	0.241297
H	0.441135	-0.908137	-0.237791	C	0.372377	-7.934026	0.844413
H	0.720831	0.848235	-0.320642	S	-0.70835	-7.349492	2.049157
H	-0.935484	0.198689	-0.301232	H	1.106753	-4.709718	0.409568
H	2.346165	0.947079	1.716475	H	1.769465	-7.059032	-0.546763
H	2.276277	-0.785438	2.120979	H	0.449912	-8.991635	0.647369
H	1.975873	0.421862	3.375168				

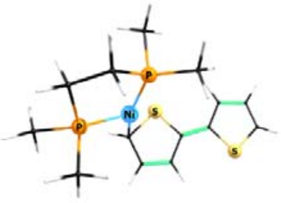

Geometry	8H
E(Hatree)	-3543.76255
Gibbs(Hatree)	-3543.48959
Enthalpy(Hatree)	-3543.41683
Entropy Correction(Hatree)	-0.0727613
# Imaginary Frequencies	0

CARTESIAN COORDINATES (ANGSTROEM)

RKS B3LYP def2-TZVP def2-TZVP/C RIJCOSX Grid4

C	-0.869047	-0.051456	-0.456126	H	-1.854238	-2.077752	-4.097622
C	-0.46022	0.004395	0.867475	H	-2.305996	-3.683185	-4.701108
C	-1.458635	-0.260202	1.807141	H	-3.550916	-2.556748	-4.106283
S	-2.956694	-0.622419	0.984934	H	-3.168852	-5.79176	-2.883472
C	-2.210789	-0.479434	-0.624539	H	-4.45373	-4.635146	-2.563873
Ni	-2.73053	-2.453632	-0.357283	H	-2.763145	-6.115498	-0.478169

P	-3.833431	-4.175996	0.48116	H	-4.498302	-6.25252	-0.708976
C	-3.657362	-5.555503	-0.766954	H	-2.571221	-5.362548	2.215195
C	-3.492903	-5.006971	-2.193996	H	-4.246563	-5.958272	2.143387
P	-2.331106	-3.546241	-2.229843	H	-3.88039	-4.392345	2.893555
C	-0.671114	-4.338309	-2.377526	H	-5.867101	-3.117283	1.280392
C	-2.533367	-2.919505	-3.952945	H	-6.01232	-3.520411	-0.425397
C	-3.612919	-5.069849	2.080871	H	-6.206232	-4.79386	0.807323
C	-5.65662	-3.88825	0.53759	C	-2.257015	-0.832781	4.156518
H	-2.832856	-0.142141	-1.445758	C	-1.903282	-0.584573	5.507875
H	-0.248849	0.241584	-1.291695	C	-0.769122	0.161531	5.63721
H	0.548411	0.257781	1.168968	S	-0.125975	0.59412	4.094095
C	-1.393138	-0.263224	3.241622	H	-3.112273	-1.423975	3.860215
H	0.065439	-3.563851	-2.598814	H	-2.464617	-0.961781	6.352337
H	-0.646502	-5.086634	-3.173517	H	-0.270301	0.477335	6.539088
H	-0.387057	-4.808626	-1.43591				

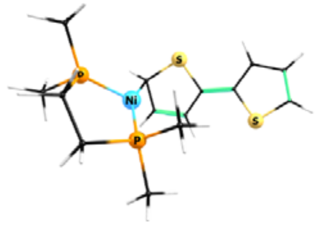

Geometry	5H
E(Hatree)	-3543.77389
Gibbs(Hatree)	-3543.49982
Enthalpy(Hatree)	-3543.42779
Entropy Correction(Hatree)	-0.0720312
# Imaginary Frequencies	0

CARTESIAN COORDINATES (ANGSTROEM)

RKS B3LYP def2-TZVP def2-TZVP/C RIJCOSX Grid4

Ni	8.169874	2.732962	14.586044	C	6.671381	3.59793	17.753134
P	6.669922	1.615422	13.428908	C	6.041097	5.261244	15.555868
P	6.615556	3.539899	15.913118	S	10.460494	7.56133	15.222555
C	11.202684	8.853521	14.362456	S	10.551928	3.879725	12.895672
H	11.136224	9.863125	14.734925	C	11.733694	6.997135	13.073821
C	11.832718	8.40285	13.238529	H	12.184087	6.451348	12.256788
H	12.362051	9.052172	12.55514	C	10.4644	4.33959	15.44239
C	11.032775	6.37581	14.079774	H	10.549555	4.835901	16.401097
C	10.730081	4.974983	14.269974	H	6.06363	2.588627	11.275826
C	10.057623	2.966546	15.308267	H	5.420208	0.938227	11.408736
H	10.163919	2.270192	16.133009	H	7.142314	1.204248	11.071544
C	10.047437	2.529543	13.947185	H	5.797871	-0.701277	13.503807
H	10.348588	1.544418	13.616481	H	6.893457	-0.353436	14.866193
C	5.000545	2.129323	14.107718	H	7.55583	-0.641732	13.261691
H	4.688288	2.990718	13.509499	H	7.44232	4.307628	18.056275
H	4.251282	1.348553	13.946805	H	6.944412	2.619311	18.149502
C	5.083811	2.524101	15.5898	H	5.719072	3.914202	18.18736
H	5.169041	1.628104	16.21162	H	5.163891	5.525131	16.152236
H	4.180976	3.051609	15.911679	H	5.797148	5.3709	14.498473
C	6.279815	1.577944	11.624724	H	6.846792	5.960419	15.783289
C	6.719765	-0.194839	13.801203				

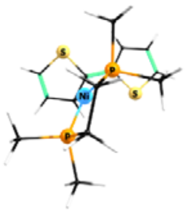

Geometry	7H
E(Hatree)	-3543.77037
Gibbs(Hatree)	-3543.4974
Enthalpy(Hatree)	-3543.42463
Entropy Correction(Hatree)	-0.0727773
# Imaginary Frequencies	0

CARTESIAN COORDINATES (ANGSTROEM)

RKS B3LYP def2-TZVP def2-TZVP/C RIJCOSX Grid4

P	-1.181109	1.762081	11.307041	H	2.268733	-0.041127	7.657246
Ni	-0.347297	-0.277319	11.327367	H	3.514464	1.064635	9.788147
P	1.161881	0.298556	9.847335	H	3.270795	-0.305739	10.900773
C	-1.491404	2.815682	12.781359	H	2.769318	1.308054	11.384482
C	-2.782172	1.933402	10.408831	C	-1.290856	-1.448807	12.663157
C	-0.005838	2.812016	10.304192	S	-0.532188	-1.348166	14.29731
C	0.657145	1.976164	9.198256	C	0.80191	-2.360505	13.80438
C	1.569318	-0.595294	8.288016	C	0.715429	-2.755149	12.518583
C	2.845123	0.62974	10.534142	C	-0.439755	-2.237774	11.8116
H	0.754008	3.174398	11.003135	C	-2.751165	-1.357218	12.599671
H	-0.504843	3.693454	9.892094	H	1.590516	-2.575772	14.508924
H	-0.056794	1.79056	8.390716	H	1.448393	-3.396964	12.047083
H	1.50735	2.502128	8.75435	H	-0.842881	-2.777638	10.960425
H	-2.239151	2.326625	13.406937	C	-3.609422	-0.685852	13.439631
H	-1.849982	3.812214	12.511576	C	-4.976056	-0.791916	13.066116
H	-0.574963	2.906043	13.365928	C	-5.163196	-1.549756	11.948121
H	-2.730559	1.420132	9.44851	S	-3.665698	-2.157579	11.347547
H	-3.051856	2.979826	10.244803	H	-3.267796	-0.134488	14.30525
H	-3.560504	1.448896	11.000801	H	-5.784531	-0.323151	13.611235
H	0.650892	-0.777118	7.728578	H	-6.087273	-1.795862	11.450388
H	2.008015	-1.563311	8.535962				

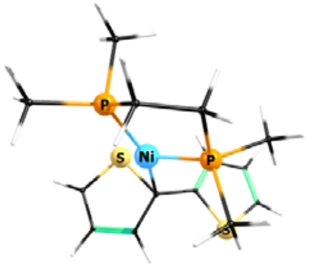

Geometry	9H
E(Hatree)	-3543.755
Gibbs(Hatree)	-3543.4822
Enthalpy(Hatree)	-3543.409
Entropy Correction(Hatree)	-0.0731549
# Imaginary Frequencies	-17.3

CARTESIAN COORDINATES (ANGSTROEM)

RKS B3LYP def2-TZVP def2-TZVP/C RIJCOSX Grid4

P	-1.677696	2.02747	11.050111	H	-2.022906	-0.408892	6.247937
Ni	-2.183677	-0.109769	10.768607	H	0.453228	-0.242458	7.285668
P	-1.595571	0.006454	8.650945	H	0.270973	-1.548969	8.484186
C	-0.385593	2.616979	12.223257	H	0.816412	0.040974	9.001866
C	-3.029083	3.249287	11.338547	C	-2.295884	-0.427336	13.684193
C	-0.995711	2.6205	9.411201	S	-3.053966	0.661386	14.814321

C	-1.579217	1.821777	8.236188	C	-1.652368	0.714973	15.821362
C	-2.463823	-0.724313	7.196938	C	-0.664385	-0.082846	15.326521
C	0.152889	-0.478186	8.310146	C	-1.027694	-0.731193	14.116502
H	0.088423	2.48277	9.449699	S	-2.38236	-2.263609	11.511765
H	-1.176527	3.691956	9.285586	H	0.293685	-0.215787	15.810904
H	-2.620122	2.110017	8.061322	H	-0.375474	-1.406865	13.580794
H	-1.029309	2.021456	7.311367	C	-3.006343	-0.878549	12.483357
H	-0.743806	2.471396	13.243209	C	-4.421757	-0.887369	12.337292
H	-0.132183	3.670319	12.075113	C	-4.921415	-1.946776	11.569682
H	0.513041	2.011974	12.097489	C	-3.963144	-2.818896	11.102046
H	-3.831243	3.08371	10.618326	H	-5.055326	-0.214962	12.89981
H	-2.669327	4.277023	11.244274	H	-5.978996	-2.104595	11.398825
H	-3.442618	3.102573	12.337279	H	-4.089383	-3.698616	10.49376
H	-3.51449	-0.430789	7.218571	H	-1.649085	1.312116	16.718573
H	-2.41977	-1.812539	7.264758				

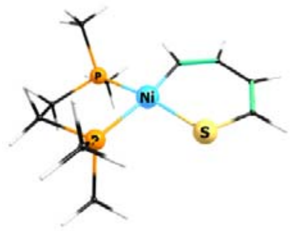

Geometry	4H (A)
E(Hatree)	-2992.02135
Gibbs(Hatree)	-2991.78727
Enthalpy(Hatree)	-2991.72460
Entropy Correction(Hatree)	-0.0626716
# Imaginary Frequencies	0

CARTESIAN COORDINATES (ANGSTROEM)

RKS B3LYP def2-TZVP def2-TZVP/C RIJCOSX Grid4

C	-0.06137	0.103307	-0.009459	H	2.228003	-0.9579	1.860731
P	0.010806	0.002253	1.818043	H	2.321004	0.790834	1.535747
C	1.821923	0.019184	2.125565	H	2.021417	0.192694	3.183844
Ni	-1.259298	-1.501935	2.974559	H	0.24869	2.446566	2.136463
C	-2.478298	-2.603356	3.945381	H	-1.394201	1.965747	1.739291
C	-2.6108	-3.951497	3.989518	H	-1.407088	2.602103	4.1389
C	-1.856112	-4.944522	3.285167	H	-0.000775	1.593706	4.449213
C	-0.879416	-4.685843	2.385484	H	-0.807058	-0.301522	6.281713
H	-0.385472	-5.509099	1.879294	H	-2.077507	0.930804	6.528692
C	-0.524381	1.703171	2.347698	H	-2.4985	-0.784202	6.337142
C	-0.900783	1.682616	3.834525	H	-3.872684	1.626557	4.673652
P	-1.943103	0.186625	4.19419	H	-3.836546	1.119994	2.968158
C	-1.818883	0.003024	6.013316	H	-4.358405	-0.019419	4.210346
S	-0.297896	-3.140854	1.906399	H	-2.112375	-5.982424	3.475318
C	-3.666892	0.795542	3.995404	H	-3.396202	-4.353861	4.630424
H	-1.088119	0.28904	-0.326493	H	-3.210039	-2.103044	4.583418
H	0.260674	-0.85004	-0.430027				
H	0.581023	0.900318	-0.389537				

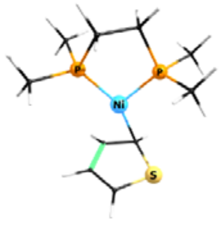

Geometry	3H (A)
E(Hatree)	-2992.01443

Gibbs(Hatree)	-2991.78348
Enthalpy(Hatree)	-2991.71909
Entropy Correction(Hatree)	-0.0643926
# Imaginary Frequencies	0

CARTESIAN COORDINATES (ANGSTROEM)

RKS B3LYP def2-TZVP def2-TZVP/C RIJCOSX Grid4

P	-1.573261	1.355626	10.920034	H	-2.418917	-0.499188	9.581013
Ni	0.051134	0.358966	11.993902	H	-3.528101	0.886563	9.473661
P	1.319003	0.399501	10.209286	H	-3.496207	-0.138788	10.926566
C	-0.257503	0.175284	13.981961	H	2.635261	-0.701165	8.426639
C	1.015877	-0.351096	13.596108	H	1.336326	-1.747555	9.044587
C	-2.5669	2.761669	11.576343	H	2.804939	-1.513446	9.997112
C	-2.883768	0.311318	10.143589	H	3.274315	1.674292	9.396756
C	-0.750813	2.133315	9.430767	H	3.455574	1.147963	11.090382
C	0.308669	1.186874	8.844757	H	2.41829	2.514377	10.713199
C	2.100568	-1.021485	9.324696	S	2.321715	0.662413	14.265452
C	2.75797	1.54301	10.350899	C	1.168856	1.799711	14.91325
H	-0.276743	3.050516	9.793178	C	-0.1121	1.42171	14.712997
H	-1.48473	2.429869	8.675335	H	1.520502	2.699765	15.394058
H	-0.180156	0.372105	8.302381	H	-0.956319	1.996878	15.071205
H	0.950539	1.70448	8.125956	H	-1.122944	-0.45917	14.138659
H	-3.225798	2.391282	12.36349	H	1.245794	-1.407076	13.529098
H	-3.173829	3.247	10.807323				
H	-1.89581	3.496872	12.022428				

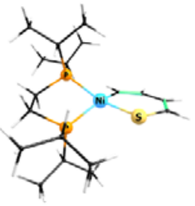

Geometry	iPr-4 (D)
E(Hatree)	-3306.37056
Gibbs(Hatree)	-3305.92096
Enthalpy(Hatree)	-3305.83529
Entropy Correction(Hatree)	-0.0856716
# Imaginary Frequencies	0

CARTESIAN COORDINATES (ANGSTROEM)

RKS B3LYP def2-TZVP def2-TZVP/C RIJCOSX Grid4

C	-0.45095	0.409342	0.058607	H	-3.031639	0.929585	2.571598
P	-0.041221	0.019787	1.853356	C	-4.529093	-0.218303	3.537065
C	1.807548	-0.196379	2.121356	H	-1.636447	-6.173172	3.537418
Ni	-1.114237	-1.614639	3.122312	H	-1.437655	-4.757756	5.453561
C	-1.299497	-2.895745	4.507343	H	-1.272454	-2.482483	5.514319
C	-1.417126	-4.24553	4.490362	H	-3.260942	-1.066799	7.708407
C	-1.498765	-5.112654	3.352028	H	-3.479569	-1.676299	6.067747
C	-1.376077	-4.706844	2.067766	H	-4.200035	-0.132691	6.549494
H	-1.405726	-5.441158	1.269347	H	-5.01143	-0.349796	4.504726
C	-0.319533	1.662451	2.694781	H	-4.182541	-1.193784	3.194625
C	-0.609892	1.472756	4.185064	H	-5.290138	0.136854	2.837807
P	-1.826425	0.090881	4.397855	H	2.21101	-1.959815	0.891162

C	-2.04281	-0.027869	6.257377	H	1.665808	-2.339745	2.514094
S	-1.106518	-3.101321	1.517486	H	3.312697	-1.739987	2.258001
C	-3.379597	0.797844	3.601055	H	-0.381179	-0.491369	-1.900151
H	-1.54424	0.328122	0.083547	H	-0.221926	-1.668634	-0.593365
C	0.055269	-0.664799	-0.912683	H	1.140949	-0.623763	-1.023411
C	-0.101409	1.816734	-0.446558	H	-0.515053	1.947816	-1.450643
C	2.271597	-1.646106	1.934816	H	0.975143	1.973568	-0.515338
C	2.728307	0.787981	1.393101	H	-0.517354	2.607898	0.176462
H	1.876096	0.025596	3.194028	H	2.74981	0.598564	0.318563
H	0.529615	2.330354	2.541763	H	3.749918	0.671289	1.765535
H	-1.176032	2.138689	2.215959	H	2.444093	1.829877	1.549746
H	-0.950992	2.405382	4.637227	H	-4.236335	2.13193	5.115781
H	0.295605	1.169636	4.718086	H	-4.675056	2.518352	3.459047
H	-1.194347	-0.661079	6.539423	H	-3.070869	2.92462	4.050048
C	-1.916319	1.28343	7.045033	H	-2.036287	1.069032	8.111044
C	-3.323627	-0.773612	6.65708	H	-2.686881	2.005616	6.774336
C	-3.856358	2.168315	4.093594	H	-0.945004	1.761459	6.914495

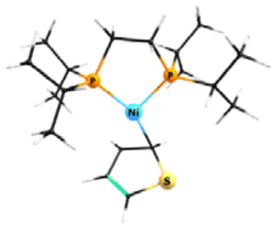

Geometry	iPr-3 (D)
E(Hatree)	-3306.37054
Gibbs(Hatree)	-3305.92257
Enthalpy(Hatree)	-3305.83779
Entropy Correction(Hatree)	-0.08478387
# Imaginary Frequencies	0

CARTESIAN COORDINATES (ANGSTROEM)

RKS B3LYP def2-TZVP def2-TZVP/C RIJCOSX Grid4

Ni	8.204282	2.914018	14.707272	C	5.541789	-0.814023	13.035669
P	6.812716	1.801007	13.399967	H	4.581367	-0.301806	12.96556
P	6.540391	3.572106	16.02441	H	5.362286	-1.778803	13.520123
C	10.731126	5.23306	14.035625	H	5.888601	-1.021156	12.022106
C	10.080883	3.396085	15.350213	H	6.194443	0.074315	14.85835
H	10.26668	2.820368	16.251077	C	6.551008	2.930214	17.793549
C	10.07663	2.778056	14.05822	H	6.702985	1.861623	17.593631
H	10.416488	1.769006	13.862609	C	5.276719	3.056689	18.636941
C	5.12718	2.491563	13.826284	H	5.108213	4.079417	18.973023
H	5.038542	3.4412	13.290618	H	5.371562	2.434613	19.532267
H	4.310291	1.856675	13.475578	H	4.384923	2.725123	18.103537
C	5.006935	2.732097	15.340502	C	7.785182	3.408887	18.566028
H	4.908051	1.775205	15.859555	H	8.698909	3.223425	18.002504
H	4.100318	3.298385	15.564307	H	7.859931	2.879473	19.520028
C	6.907533	2.025841	11.5368	H	7.735115	4.477149	18.790413
H	7.142348	3.096017	11.481663	C	6.012963	5.38249	15.928603
C	8.113156	1.283923	10.946782	C	4.816133	5.837398	16.770083
H	9.017575	1.468367	11.526489	H	3.96336	5.161849	16.690386
H	8.296508	1.624082	9.924192	H	4.481629	6.823569	16.434433
H	7.942995	0.205768	10.907991	H	5.082362	5.929947	17.824332

C	5.641981	1.79006	10.706135	C	7.192577	6.345964	16.083678
H	5.3599	0.737387	10.673103	H	7.552862	6.378451	17.11517
H	5.817158	2.115771	9.676121	H	6.880099	7.358861	15.812673
H	4.787635	2.354198	11.081825	H	8.028446	6.06258	15.44583
C	6.577032	-0.016429	13.8346	H	5.706866	5.42782	14.875255
C	7.90903	-0.771857	13.929401	S	10.568524	3.980513	12.830527
H	8.347723	-0.945254	12.945079	C	10.473295	4.790774	15.284507
H	7.753317	-1.749003	14.395798	H	10.547624	5.423862	16.159374
H	8.635324	-0.220738	14.527458	H	10.982998	6.237216	13.7299

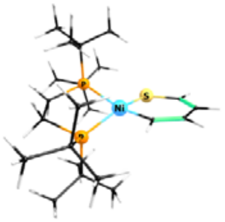

Geometry	tBu-4 (E)
E(Hatree)	-3463.53894
Gibbs(Hatree)	-3462.97917
Enthalpy(Hatree)	-3462.88629
Entropy Correction(Hatree)	-0.09287256
# Imaginary Frequencies	0

CARTESIAN COORDINATES (ANGSTROEM)

RKS B3LYP def2-TZVP def2-TZVP/C RIJCOSX Grid4

C	-0.107733	0.3523	-0.144537	H	-1.716342	0.511588	-1.573093
P	0.026648	-0.002032	1.743577	H	-2.193517	1.019868	0.036405
C	1.863756	-0.281544	2.225721	H	2.946478	-0.421597	4.086147
Ni	-1.269918	-1.558748	2.974478	H	1.232632	-0.782442	4.253452
C	-1.81441	-2.883627	4.222548	H	1.778395	0.898935	4.078238
C	-2.000441	-4.222372	4.128947	H	0.443853	0.249672	6.089923
C	-1.908356	-5.050137	2.963262	H	-0.269301	-1.360264	6.212915
C	-1.567699	-4.59535	1.738201	H	-0.19433	-0.299478	7.629599
H	-1.500467	-5.287192	0.904922	H	-3.382484	0.191549	1.783778
C	-0.410064	1.684526	2.41251	H	-3.300174	1.918088	2.187509
C	-0.989861	1.703627	3.828618	H	-4.858246	1.113249	2.080457
P	-1.971732	0.194576	4.321212	H	-2.562468	-0.556784	8.0522
C	-1.744265	0.272646	6.24042	H	-2.895069	-1.56463	6.647192
S	-1.157414	-2.979155	1.322371	H	-3.805178	-0.078976	6.905959
C	-3.790359	0.620975	3.889798	H	-4.737084	-0.943911	5.12273
C	-1.602922	0.283166	-0.508629	H	-4.291747	-1.471276	3.504884
C	0.640843	-0.710868	-0.964098	H	-5.682546	-0.399612	3.739432
C	0.394411	1.752221	-0.549252	H	2.259164	-1.957411	0.840574
C	2.277092	-1.730473	1.904323	H	1.626514	-2.448195	2.401226
C	2.853302	0.702538	1.588144	H	3.299112	-1.88765	2.264338
C	1.945815	-0.129297	3.75287	H	0.42944	-0.551457	-2.025669
H	0.441884	2.365382	2.353279	H	0.318466	-1.719137	-0.705406
H	-1.155274	2.07195	1.720306	H	1.720943	-0.645876	-0.835962
H	-1.581674	2.609468	3.97365	H	0.233975	1.874449	-1.625125
H	-0.17408	1.75662	4.546957	H	1.454157	1.902424	-0.353933
C	-0.358931	-0.327654	6.548038	H	-0.158516	2.551744	-0.05741
C	-1.74653	1.708011	6.803262	H	2.959339	0.550793	0.514874
C	-2.819482	-0.534227	6.988826	H	3.840311	0.551817	2.03644

C	-4.385352	1.804751	4.666458	H	2.575323	1.743943	1.763491
C	-3.818861	0.980727	2.3947	H	-4.534828	1.583411	5.721809
C	-4.669116	-0.628047	4.083101	H	-5.366923	2.041328	4.243834
H	-2.117706	-6.109105	3.075843	H	-3.775101	2.705673	4.584391
H	-2.253037	-4.756086	5.046539	H	-1.500506	1.658084	7.868818
H	-1.984316	-2.510213	5.22697	H	-2.71172	2.201451	6.711368
H	-2.021746	-0.704701	-0.318711	H	-0.99493	2.344071	6.339294

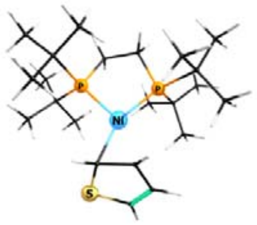

Geometry	tBu-3 (E)
E(Hatree)	-3463.54185
Gibbs(Hatree)	-3462.98649
Enthalpy(Hatree)	-3462.89147
Entropy Correction(Hatree)	-0.09501845
# Imaginary Frequencies	0

CARTESIAN COORDINATES (ANGSTROEM)

RKS B3LYP def2-TZVP def2-TZVP/C RIJCOSX Grid4

Ni	8.150218	2.801155	14.762477	H	6.210164	-0.935316	11.641811
P	6.743782	1.697462	13.434912	C	6.185155	-0.500461	15.074433
P	6.499748	3.574718	16.056831	H	6.745126	0.058397	15.819986
C	10.888114	4.944097	14.272411	H	6.31743	-1.56479	15.293938
C	10.029023	3.135043	15.50193	H	5.124043	-0.27042	15.185397
H	10.119136	2.528187	16.397396	C	6.547363	3.117804	17.9262
C	10.047422	2.546034	14.196831	C	7.042572	1.660042	17.995647
H	10.328878	1.520986	13.993391	H	6.282886	0.95575	17.655103
C	5.029431	2.24092	13.98356	H	7.273976	1.406389	19.035111
H	4.775169	3.086195	13.344705	H	7.943612	1.509012	17.40054
H	4.279561	1.471311	13.7837	C	5.207551	3.191912	18.677094
C	4.953554	2.68404	15.46006	H	4.842193	4.208796	18.799096
H	4.838078	1.808865	16.098576	H	5.335283	2.768561	19.679279
H	4.061685	3.295392	15.617625	H	4.427518	2.609514	18.184281
C	6.715678	2.116525	11.560227	C	7.589993	3.99683	18.635328
C	6.953292	3.635392	11.463504	H	8.541063	4.001345	18.101097
H	6.115109	4.205475	11.864711	H	7.772071	3.601287	19.639416
H	7.055791	3.915592	10.410072	H	7.254828	5.027782	18.746696
H	7.862623	3.939376	11.982567	C	6.039721	5.43119	15.851056
C	7.897282	1.418422	10.867284	C	4.968189	5.967003	16.809242
H	8.836833	1.627581	11.380148	H	4.061484	5.359721	16.80482
H	7.989326	1.795387	9.844074	H	4.683713	6.97872	16.501638
H	7.770555	0.337898	10.805227	H	5.3305	6.033206	17.834644
C	5.405758	1.800521	10.818434	C	7.320029	6.272095	15.982741
H	5.175183	0.738521	10.785136	H	7.724169	6.263706	16.994511
H	5.489728	2.149797	9.783764	H	7.094031	7.3118	15.723948
H	4.551986	2.31797	11.258284	H	8.100686	5.916058	15.31092
C	6.706184	-0.211605	13.65642	C	5.531279	5.607889	14.409828
C	8.151718	-0.734352	13.604698	H	6.254732	5.235778	13.686627
H	8.614961	-0.607059	12.626928	H	5.382863	6.674056	14.211957

H	8.162225	-1.802312	13.847257	H	4.574509	5.112988	14.238536
H	8.773593	-0.220368	14.337584	S	10.698719	3.733668	13.029586
C	5.823415	-0.973263	12.659323	C	10.528428	4.495267	15.492986
H	4.799063	-0.595737	12.647659	H	10.598063	5.103054	16.385932
H	5.777014	-2.028342	12.948934	H	11.23014	5.932544	14.006634

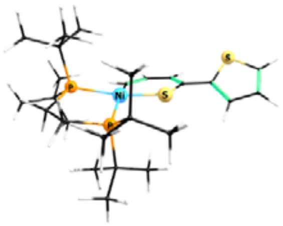

Geometry	6<br>Complex **6**
E(Hatree)	-4015.29664
Gibbs(Hatree)	-4014.69448
Enthalpy(Hatree)	-4014.59347
Entropy Correction(Hatree)	-0.10100508
# Imaginary Frequencies	0

CARTESIAN COORDINATES (ANGSTROEM)

RKS B3LYP def2-TZVP def2-TZVP/C RIJCOSX Grid4

C	-0.204901	0.352931	-0.135758	H	1.800485	0.939282	4.030688
P	-0.00524	0.016076	1.751599	H	0.456175	0.11764	6.0482
C	1.846785	-0.244487	2.178425	H	-0.357209	-1.439262	6.216849
Ni	-1.305971	-1.53234	3.011396	H	-0.163217	-0.370958	7.61717
C	-1.983304	-2.839879	4.201356	H	-3.461173	0.302411	1.851705
C	-2.221474	-4.168865	4.076375	H	-3.305597	2.021476	2.265525
C	-2.047724	-5.009091	2.933905	H	-4.893809	1.275725	2.18892
C	-1.540243	-4.603709	1.740133	H	-2.535524	-0.533283	8.09089
C	-1.374978	-5.528146	0.61226	H	-2.96608	-1.490927	6.678543
C	-0.432632	1.70238	2.424708	H	-3.7793	0.040222	6.99105
C	-0.97805	1.7228	3.854346	H	-4.788387	-0.81094	5.206762
P	-1.989156	0.236983	4.356581	H	-4.392597	-1.340551	3.575941
C	-1.71917	0.283234	6.272086	H	-5.735539	-0.21806	3.845599
S	-1.038383	-2.972567	1.412602	H	2.220647	-1.92065	0.789156
C	-3.803761	0.727331	3.969289	H	1.653076	-2.413915	2.373234
C	-1.709084	0.26499	-0.454949	H	3.309176	-1.824645	2.175448
C	0.531253	-0.702857	-0.976186	H	0.285766	-0.544953	-2.030517
C	0.268858	1.755328	-0.563995	H	0.227383	-1.714584	-0.710333
C	2.272628	-1.687791	1.850509	H	1.61394	-0.624498	-0.88138
C	2.803459	0.748676	1.505564	H	0.077485	1.86567	-1.636025
C	1.969497	-0.087705	3.702263	H	1.331703	1.919027	-0.399502
H	0.416753	2.384207	2.34477	H	-0.279838	2.552539	-0.063957
H	-1.193567	2.087659	1.748977	H	2.874029	0.592193	0.430087
H	-1.543442	2.641995	4.019588	H	3.806698	0.611	1.920314
H	-0.145163	1.750831	4.553824	H	2.519727	1.78766	1.685413
C	-0.365837	-0.399552	6.542224	H	-4.470681	1.698832	5.827489
C	-1.632012	1.71183	6.847388	H	-5.314312	2.206382	4.372897
C	-2.820383	-0.473427	7.036183	H	-3.690418	2.801947	4.685201
C	-4.334414	1.925215	4.7715	H	-1.387115	1.640271	7.911851
C	-3.853184	1.102333	2.478449	H	-2.566314	2.262242	6.761477
C	-4.725834	-0.488843	4.1687	H	-0.844675	2.305185	6.386204

H	-2.301771	-6.05826	3.04596	C	-0.337177	-5.63416	-0.281755
H	-2.585267	-4.691172	4.962558	C	-0.526746	-6.649435	-1.255948
H	-2.214541	-2.471299	5.195588	C	-1.717616	-7.304319	-1.113464
H	-2.112805	-0.724236	-0.240631	S	-2.613505	-6.681872	0.215472
H	-1.855157	0.478727	-1.518403	H	0.540395	-5.006961	-0.220856
H	-2.29012	1.004015	0.096885	H	0.196422	-6.890292	-2.023302
H	2.981102	-0.370452	4.009455	H	-2.11256	-8.113598	-1.706603
H	1.275914	-0.747101	4.222626				

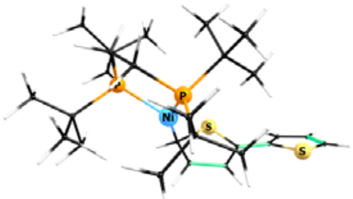

Geometry	5<br>complex **5**
E(Hatree)	-4015.29664
Gibbs(Hatree)	-4014.69602
Enthalpy(Hatree)	-4014.59704
Entropy Correction(Hatree)	-0.09897216
# Imaginary Frequencies	0

CARTESIAN COORDINATES (ANGSTROEM)

RKS B3LYP def2-TZVP def2-TZVP/C RIJCOSX Grid4

Ni	8.068866	2.890385	14.443751	H	6.098391	-2.186841	13.312137
P	6.588101	1.666957	13.284698	H	6.152823	-1.210621	11.850799
P	6.514078	3.635527	15.865733	C	6.624844	-0.392917	15.175931
C	12.074622	8.611847	15.546934	H	7.20612	0.3178	15.759907
H	12.243757	9.480805	16.162018	H	6.94779	-1.398854	15.463728
C	12.502861	8.37937	14.272164	H	5.573681	-0.294074	15.451667
H	13.084113	9.090499	13.701833	C	6.729628	3.408471	17.764058
C	11.402523	6.357873	14.689582	C	7.390937	2.029854	17.959283
C	10.88395	5.013225	14.56585	H	6.716984	1.213986	17.696413
C	10.007167	2.899465	15.141483	H	7.657242	1.901884	19.013187
H	10.077465	2.023772	15.778392	H	8.298548	1.930752	17.365477
C	9.917651	2.780462	13.719827	C	5.432944	3.421931	18.592097
H	10.178307	1.892406	13.159781	H	4.959226	4.400544	18.62306
C	4.923384	2.038307	14.078568	H	5.669752	3.137925	19.622998
H	4.460906	2.781801	13.43074	H	4.699011	2.703494	18.224446
H	4.268107	1.164196	14.052703	C	7.694853	4.477142	18.302703
C	4.994065	2.593965	15.51506	H	8.606299	4.536216	17.707604
H	5.030638	1.765901	16.222123	H	7.981928	4.223566	19.327853
H	4.079033	3.146119	15.742468	H	7.241956	5.46782	18.326784
C	6.149141	1.863769	11.416544	C	5.946618	5.436922	15.49922
C	6.114554	3.377903	11.135706	C	4.967643	6.041894	16.512982
H	5.315843	3.876263	11.684211	H	4.07892	5.422859	16.649093
H	5.917212	3.541683	10.071309	H	4.630187	7.015774	16.143054
H	7.058748	3.86235	11.388609	H	5.423315	6.209254	17.487868
C	7.269445	1.250577	10.560612	C	7.197862	6.322953	15.382539
H	8.24933	1.63454	10.851762	H	7.715479	6.451137	16.331824
H	7.110735	1.512738	9.510257	H	6.903455	7.316371	15.028548
H	7.29668	0.1629	10.621644	H	7.911091	5.910055	14.668015

C	4.784801	1.295456	10.985941	C	5.259688	5.438792	14.123962
H	4.725415	0.212659	11.059832	H	5.916954	5.039057	13.355068
H	4.605792	1.563801	9.939074	H	5.015208	6.4697	13.848685
H	3.964929	1.719951	11.565885	H	4.325917	4.875061	14.118196
C	6.858739	-0.200704	13.669012	S	11.179327	7.275609	16.158135
C	8.331921	-0.553567	13.39961	S	10.480202	4.31146	12.991616
H	8.621614	-0.414623	12.358454	C	12.120042	7.103828	13.783701
H	8.507266	-1.601995	13.662889	H	12.387704	6.72887	12.805485
H	8.99326	0.058757	14.01155	C	10.587015	4.141737	15.566713
C	5.942814	-1.176901	12.918861	H	10.790464	4.368011	16.60605
H	4.886523	-0.936661	13.052741				

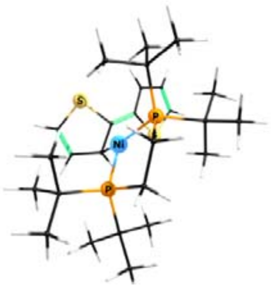

Geometry	7<br>Complexe **7**
E(Hatree)	-4015.2911
Gibbs(Hatree)	-4014.6906
Enthalpy(Hatree)	-4014.5893
Entropy Correction(Hatree)	-0.1012875
# Imaginary Frequencies	-16.2

CARTESIAN COORDINATES (ANGSTROEM)

RKS B3LYP def2-TZVP def2-TZVP/C RIJCOSX Grid4

Ni	-1.232208	1.42237	2.58538	H	4.637437	-0.392452	2.657892
P	-0.240049	3.46157	2.903017	H	2.726742	0.331699	1.039549
P	-3.267819	2.214413	3.150504	H	-5.205354	0.439887	1.517011
C	-4.205391	1.38654	4.618001	H	-3.785708	0.840856	0.556059
C	-3.136691	0.900654	5.614482	H	-5.379653	1.431948	0.074861
C	-4.973954	0.143465	4.136117	H	-6.475057	2.345371	2.731685
C	-5.164488	2.315915	5.381337	H	-6.478069	3.340686	1.282802
C	-4.530306	2.528617	1.728511	H	-5.8079	3.982377	2.775812
C	-4.732008	1.226152	0.932994	H	-4.539158	3.620447	-0.129228
C	-5.895264	3.077247	2.171568	H	-2.912257	3.248241	0.434428
C	-3.906946	3.549422	0.760529	H	-3.850161	4.548963	1.193059
C	0.758687	3.736151	4.543582	H	0.271966	1.709573	5.220898
C	0.203218	2.738553	5.573837	H	-0.836789	2.947811	5.822262
C	2.249484	3.418749	4.353765	H	0.78071	2.821592	6.500299
C	0.634422	5.146617	5.14666	H	2.757079	4.167411	3.747653
C	0.69785	4.328157	1.455773	H	2.414228	2.437457	3.906612
C	1.937138	3.507843	1.056015	H	2.732539	3.423124	5.335639
C	1.112683	5.779499	1.740287	H	0.943059	5.935099	4.462433
C	-0.232729	4.321995	0.2273	H	1.281482	5.20837	6.027695
C	-1.708882	4.603512	3.134924	H	-0.379387	5.366357	5.484436
C	-2.912292	3.931464	3.820003	H	-2.415781	0.2332	5.142853
H	-1.994837	4.92284	2.136588	H	-3.629527	0.355147	6.4254
H	-1.434484	5.509441	3.674997	H	-2.587603	1.725905	6.066802
H	-3.787958	4.582465	3.758103	H	-4.338445	-0.521251	3.549408

H	-2.700224	3.806911	4.883545	H	-5.851745	0.393529	3.541904
S	-0.02819	-0.089262	0.075527	H	-5.323986	-0.416596	5.00845
C	-1.55651	-0.93168	0.029811	H	-5.637673	1.748708	6.190042
C	-2.075864	-1.13011	1.256679	H	-5.959102	2.720253	4.758809
C	-1.281567	-0.583988	2.338382	H	-4.638446	3.152247	5.843574
C	-0.021469	-0.047979	1.886077	H	2.382059	3.957998	0.162836
C	1.280407	-0.296993	2.517182	H	1.667909	2.481642	0.809893
S	1.398728	-1.043303	4.088678	H	2.704733	3.485663	1.823849
C	3.123131	-1.018402	4.063169	H	1.564364	6.208084	0.839269
C	3.586713	-0.48596	2.898057	H	1.855607	5.853261	2.534105
C	2.544758	-0.082787	2.020828	H	0.260704	6.407549	2.00784
H	-1.997573	-1.184391	-0.922594	H	-0.578892	3.31523	-0.010846
H	-3.017496	-1.637194	1.423796	H	0.326358	4.695566	-0.635458
H	-1.361706	-1.031834	3.325147	H	-1.099665	4.971522	0.348309
H	3.684366	-1.401051	4.899747				

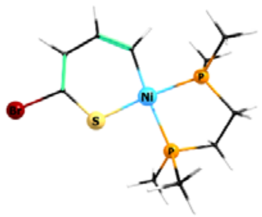

Geometry	$\mathbf{4Br_H}$
E(Hatree)	-5574.58382
Gibbs(Hatree)	-5574.36314
Enthalpy(Hatree)	-5574.29564
Entropy Correction(Hatree)	-0.06750
# Imaginary Frequencies	0.00000

CARTESIAN COORDIANTES(ANGSTROEM)

RKS B3LYP def2-TZVP def2-TZVP/C RIJCOSX Grid4

C	0.12291	-0.00343	0.02527	H	0.79749	0.77797	-0.32897
P	0.00787	-0.00263	1.85070	H	2.21982	-0.92948	2.14051
C	1.77796	0.04705	2.34277	H	2.32516	0.81167	1.78791
Ni	-1.29295	-1.47549	3.00131	H	1.87156	0.24648	3.41116
C	-2.36921	-2.59934	4.10895	H	0.13980	2.46537	2.00699
C	-2.51678	-3.94632	4.08781	H	-1.47312	1.88122	1.61870
C	-1.94132	-4.90432	3.18791	H	-1.58427	2.66174	3.96234
C	-1.15207	-4.58480	2.13987	H	-0.11057	1.79851	4.36751
Br	-0.52610	-6.05204	1.00496	H	-0.69717	-0.10498	6.26787
C	-0.60299	1.70332	2.25632	H	-1.99587	1.09703	6.50460
C	-1.00294	1.76462	3.73689	H	-2.36724	-0.63665	6.43566
P	-1.94585	0.23061	4.21289	H	-3.93324	1.58058	4.70229
C	-1.73062	0.14916	6.03140	H	-3.92189	1.00569	3.01772
S	-0.61405	-3.03968	1.63405	H	-4.34330	-0.10772	4.32059
C	-3.70512	0.73394	4.05137	H	-2.17826	-5.94754	3.35739
H	-0.86621	0.14894	-0.40636	H	-3.16605	-4.39206	4.84234
H	0.49405	-0.97464	-0.30476	H	-2.96809	-2.13823	4.89613

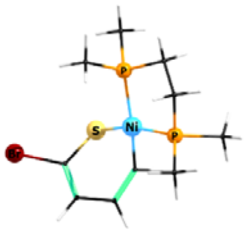

Geometry	TS1Br
E(Hatree)	-5574.58382
Gibbs(Hatree)	-5574.36314
Enthalpy(Hatree)	-5574.26462
Entropy Correction(Hatree)	-0.06616
# Imaginary Frequencies	-310.05

CARTESIAN COORDIANTES(ANGSTROEM)

RKS B3LYP def2-TZVP def2-TZVP/C RIJCOSX Grid4

C	-0.14925	0.03310	0.06062	H	4.96975	-1.28469	3.38644
P	-0.07799	-0.07527	1.89753	H	2.89796	-0.53414	5.83518
C	-1.86362	-0.29604	2.29105	H	3.17413	-2.13589	5.11709
Ni	1.30242	1.33356	3.00064	H	1.52375	-1.50129	5.30212
P	2.66072	-0.41942	3.42431	H	2.51726	-2.74721	2.56876
C	2.55643	-1.23572	5.07295	H	2.56621	-1.53745	1.28974
S	2.33975	3.07190	3.84503	H	0.26101	-2.47325	1.36367
C	1.37263	3.80212	5.07615	H	0.15893	-2.16917	3.08891
Br	2.20186	4.20295	6.76801	H	-1.98479	-0.54790	3.34561
C	0.07342	4.10850	4.79298	H	-2.31618	-1.08374	1.68428
C	-0.29774	3.86685	3.45047	H	-2.38958	0.64081	2.10227
C	0.54402	3.09552	2.67503	H	-0.76358	-0.76576	-0.36057
C	0.59307	-1.79749	2.15644	H	0.85445	-0.03221	-0.36076
C	2.12634	-1.77248	2.26371	H	-0.57656	0.99438	-0.22952
C	4.48068	-0.32666	3.19623	H	-0.56648	4.60460	5.50976
H	4.71282	0.00834	2.18434	H	-1.11271	4.42478	3.00082
H	4.87686	0.41553	3.89057	H	0.59212	3.27773	1.59966

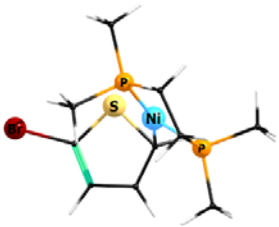

Geometry	$8Br_H$
E(Hatree)	-5574.55596
Gibbs(Hatree)	-5574.33756
Enthalpy(Hatree)	-5574.26462
Entropy Correction(Hatree)	-0.06726
# Imaginary Frequencies	-17.05

CARTESIAN COORDIANTES(ANGSTROEM)

RKS B3LYP def2-TZVP def2-TZVP/C RIJCOSX Grid4

C	-0.13191	-0.08902	-0.43432	H	-4.88297	1.36785	-2.21037
C	0.19668	0.21434	0.87764	H	-5.28802	3.08805	-2.36814
S	-1.18843	0.23437	1.90935	H	-6.47040	1.25176	0.19094
C	-2.27055	-0.19566	0.55232	H	-6.85695	2.92933	-0.23859
C	-1.47895	-0.39551	-0.60664	H	-6.33619	2.52311	1.41090

Br	1.89156	0.84301	1.49912	H	-5.16655	4.89433	-0.23843
Ni	-2.77169	1.71003	1.12717	H	-3.47792	4.69339	-0.67096
P	-4.42294	2.53523	-0.12230	H	-4.64025	4.62163	2.16920
C	-4.56470	2.38037	-1.95510	H	-3.53621	5.83445	1.53249
P	-2.42437	3.67490	2.05678	H	-3.18190	3.82808	4.36949
C	-2.28017	4.13402	3.83720	H	-2.12384	5.20707	3.97794
C	-0.92453	4.49227	1.35722	H	-1.43718	3.59750	4.27389
C	-3.80975	4.77671	1.47355	H	-0.85638	5.54489	1.64511
C	-4.24065	4.38846	0.05121	H	-0.03930	3.96340	1.71272
C	-6.19319	2.29472	0.35392	H	-0.93522	4.41676	0.26940
H	-3.14206	-0.80117	0.77750	H	-1.88660	-0.81765	-1.51481
H	-3.59240	2.55704	-2.41633	H	0.60836	-0.13339	-1.22125

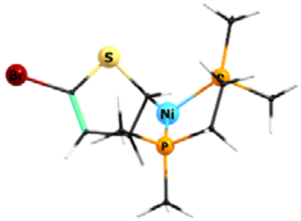

	Geometry	$\mathbf{3Br_H}$
	E(Hatree)	-5574.57211
	Gibbs(Hatree)	-5574.35211
	Enthalpy(Hatree)	-5574.28613
	Entropy Correction(Hatree)	-0.06726
	# Imaginary Frequencies	0.00

CARTESIAN COORDIANTES(ANGSTROEM)

RKS B3LYP def2-TZVP def2-TZVP/C RIJCOSX Grid4

P	-1.70775	1.38099	10.86913	H	-3.49396	1.93530	9.24285
Ni	-0.55683	-0.40689	11.41925	H	-3.88666	0.50571	10.22331
P	0.93815	0.07388	9.88254	H	2.18506	-0.48889	7.82002
C	-2.49984	2.62836	11.96730	H	0.67585	-1.41962	7.96969
C	-3.08774	1.02407	9.69055	H	2.07616	-1.85747	8.94782
C	-0.57970	2.43800	9.82611	H	3.17504	1.11505	9.75764
C	0.29004	1.54682	8.92732	H	3.06644	-0.12072	11.03558
C	1.52960	-1.01719	8.51708	H	2.36170	1.46778	11.29884
C	2.54177	0.70067	10.54573	C	-1.29347	-1.35165	13.00127
H	0.04773	3.00316	10.52185	S	-0.63525	-0.79458	14.57035
H	-1.14593	3.16630	9.23825	C	0.84653	-1.68478	14.30113
H	-0.31386	1.14212	8.10978	C	0.87583	-2.36427	13.13761
H	1.10490	2.11426	8.46911	C	-0.33294	-2.19558	12.35263
H	-3.27870	2.13624	12.55231	H	-2.36035	-1.53808	12.99950
H	-2.94528	3.45198	11.40367	Br	2.28259	-1.54503	15.55480
H	-1.76148	3.02646	12.66462	H	1.71978	-2.95841	12.81570
H	-2.73696	0.36359	8.89752	H	-0.64125	-2.96626	11.65254

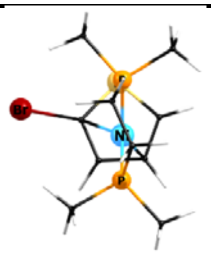

Geometry	**TS2Br**
E(Hatree)	-5574.55132
Gibbs(Hatree)	-5574.33225
Enthalpy(Hatree)	-5574.26546
Entropy Correction(Hatree)	-0.06679
# Imaginary Frequencies	-167.23

CARTESIAN COORDIANTES(ANGSTROEM)

RKS B3LYP def2-TZVP def2-TZVP/C RIJCOSX Grid4

P	-2.15975	1.41276	10.87389	H	-4.14691	2.10029	9.56230
Ni	-0.96523	-0.33315	11.41818	H	-4.46795	0.71129	10.62541
P	0.53108	0.10310	9.86199	H	0.19216	-1.53952	8.08208
C	-2.68868	2.69899	12.08105	H	1.67382	-1.86504	8.98282
C	-3.74015	1.16194	9.94831	H	1.65680	-0.58309	7.75473
C	-1.11395	2.38666	9.67604	H	2.70554	1.20129	9.39123
C	-0.25014	1.44685	8.82251	H	2.78948	0.09359	10.77952
C	1.06907	-1.07417	8.53551	H	2.04864	1.67784	10.97400
C	2.17711	0.84659	10.28015	C	-2.41591	-2.01043	12.26796
H	-0.47514	3.03618	10.28137	S	-2.77230	-1.10636	13.74336
H	-1.72853	3.03876	9.04838	C	-1.06104	-0.65308	13.77122
H	-0.87728	0.93237	8.08851	C	-0.26128	-1.51416	13.02105
H	0.50445	2.00475	8.25985	C	-1.06285	-2.30357	12.13525
H	-3.35831	2.23775	12.80884	Br	-0.40216	0.41004	15.23045
H	-3.20559	3.53055	11.59517	H	0.80721	-1.60647	13.13882
H	-1.81983	3.07819	12.62000	H	-0.66595	-3.04295	11.45529
H	-3.58456	0.47078	9.11965	H	-3.22394	-2.49117	11.73910

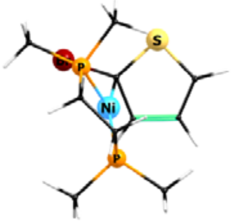

Geometry	**$3Br_X$**
E(Hatree)	-5574.57525
Gibbs(Hatree)	-5574.35551
Enthalpy(Hatree)	-5574.28918
Entropy Correction(Hatree)	-0.06633
# Imaginary Frequencies	0.00

CARTESIAN COORDIANTES(ANGSTROEM)

RKS B3LYP def2-TZVP def2-TZVP/C RIJCOSX Grid4

P	-1.57116	1.40950	10.89109	H	-1.90554	3.55481	11.97784
Ni	0.06179	0.43429	11.99517	H	-2.38907	-0.48815	9.59923
P	1.31376	0.42717	10.18113	H	-3.51491	0.88144	9.45962
C	-0.24066	0.22102	13.98104	H	-3.47108	-0.11055	10.93551
C	1.06363	-0.17895	13.53119	H	2.61142	-0.79394	8.47216
C	-2.57089	2.81026	11.53928	H	1.25755	-1.75857	9.10872

C	-2.86458	0.32926	10.14244	H	2.71162	-1.54462	10.08230
C	-0.74886	2.16085	9.39198	H	3.28900	1.64123	9.33574
C	0.30208	1.19895	8.81285	H	3.46519	1.15004	11.03932
C	2.04686	-1.05352	9.37087	H	2.45549	2.53006	10.63474
C	2.77343	1.54372	10.29397	S	2.30571	0.90555	14.22260
H	-0.26792	3.08037	9.73866	C	1.08462	1.86760	15.02218
H	-1.48466	2.45079	8.63610	C	-0.16645	1.40438	14.82143
H	-0.19389	0.38089	8.28289	H	1.38132	2.74758	15.57265
H	0.94254	1.70470	8.08501	H	-1.04132	1.86575	15.26210
H	-3.22416	2.43898	12.33044	H	-1.04367	-0.49515	14.10979
H	-3.18372	3.28259	10.76728	Br	1.59794	-2.11592	13.50976

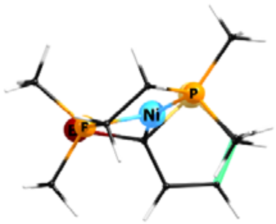

Geometry	$\mathbf{8Br_X}$
E(Hatree)	-5574.56374
Gibbs(Hatree)	-5574.34427
Enthalpy(Hatree)	-5574.28918
Entropy Correction(Hatree)	-0.06520
# Imaginary Frequencies	0.00

CARTESIAN COORDIANTES(ANGSTROEM)

RKS B3LYP def2-TZVP def2-TZVP/C RIJCOSX Grid4

C	-0.10585	-0.00034	0.03335	H	4.90574	1.35475	-2.14396
C	-0.08916	-0.02562	1.40791	H	5.45466	3.03941	-2.30276
S	1.53216	0.00847	1.99972	H	6.34958	1.12396	0.34150
C	2.24351	-0.00164	0.33042	H	6.91972	2.75787	-0.08146
C	1.16025	-0.01638	-0.57702	H	6.26903	2.42009	1.54019
H	-0.92203	-0.05160	2.09139	H	5.32830	4.89273	-0.07589
Ni	2.70348	1.73805	0.98633	H	3.68798	4.78213	-0.68958
P	4.46463	2.57225	-0.09897	H	4.53070	4.57389	2.25625
C	4.66236	2.39363	-1.91675	H	3.55561	5.85645	1.55279
P	2.30792	3.74141	1.87651	H	2.81470	3.65733	4.25448
C	2.03783	4.13053	3.65373	H	2.04589	5.20671	3.84383
C	0.91575	4.64517	1.07430	H	1.07675	3.72263	3.97095
C	3.79104	4.79069	1.47950	H	0.85490	5.68713	1.39892
C	4.35601	4.42400	0.09866	H	-0.01806	4.13736	1.32028
C	6.17160	2.19152	0.47800	H	1.03057	4.61302	-0.00956
Br	3.81225	-1.30049	-0.07304	H	1.30977	-0.13896	-1.63995
H	3.72490	2.64197	-2.41546	H	-1.02692	-0.00349	-0.53679

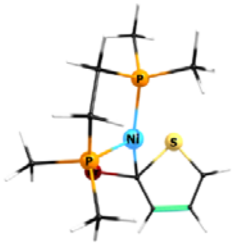

Geometry	TS3Br
E(Hatree)	-5574.55884
Gibbs(Hatree)	-5574.34080
Enthalpy(Hatree)	-5574.27440
Entropy Correction(Hatree)	-0.06520
# Imaginary Frequencies	-193.11

CARTESIAN COORDIANTES(ANGSTROEM)

RKS B3LYP def2-TZVP def2-TZVP/C RIJCOSX Grid4

C	-0.31414	0.03711	0.40422	H	6.25654	-3.87867	0.24971
C	0.24203	0.33663	1.66944	H	3.22127	-3.71884	-1.11605
C	1.57816	0.02162	1.80153	H	4.00172	-5.14180	-0.38127
S	2.25001	-0.30963	-0.10060	H	2.37273	-4.63620	0.12296
C	0.58262	-0.27630	-0.57720	H	4.62664	-5.26086	2.28031
Ni	2.65394	-1.53426	1.71833	H	5.04056	-3.80783	3.18219
P	4.05363	-3.16135	1.08832	H	3.10787	-4.87039	4.31893
C	5.77253	-2.92653	0.47923	H	2.20943	-4.94408	2.81005
Br	2.69442	1.14926	3.09355	H	-0.05662	-3.40285	3.22890
P	2.22702	-2.66266	3.62418	H	0.39340	-3.68254	4.92929
C	2.95199	-2.16092	5.24125	H	0.00826	-2.04716	4.34969
C	0.48051	-2.99474	4.08526	H	2.58240	-2.78552	6.05741
C	2.94993	-4.36128	3.36441	H	4.03940	-2.23737	5.19762
C	4.26325	-4.26955	2.56572	H	2.69786	-1.11853	5.43436
C	3.35541	-4.28205	-0.19131	H	-1.37603	0.11676	0.20315
H	6.36003	-2.39185	1.22581	H	-0.30439	0.86191	2.44024
H	5.74773	-2.31797	-0.42578	H	0.34845	-0.47036	-1.61366

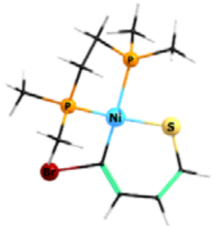

Geometry	$4Br_X$
E(Hatree)	-5574.58573
Gibbs(Hatree)	-5574.34080
Enthalpy(Hatree)	-5574.29826
Entropy Correction(Hatree)	-0.06529
# Imaginary Frequencies	0.00

CARTESIAN COORDIANTES(ANGSTROEM)

RKS B3LYP def2-TZVP def2-TZVP/C RIJCOSX Grid4

C	-0.26682	0.20016	-0.05071	H	0.33601	1.01413	-0.45787
P	-0.02079	0.02059	1.75509	H	2.15665	-1.02086	1.57793
C	1.80239	-0.04491	1.90873	H	2.27149	0.74023	1.31293
Ni	-1.29153	-1.54761	2.82571	H	2.08576	0.07775	2.95481
P	-2.13542	0.18401	4.02025	H	0.40939	2.37760	2.33672
C	-2.30371	-0.13295	5.81405	H	-1.26266	2.10156	1.87182

C	-0.43204	1.69031	2.45093	H	-1.19551	2.44367	4.35978
C	-0.83789	1.51006	3.91845	H	0.01815	1.17569	4.51105
S	0.17652	-3.02790	2.15192	H	-1.37138	-0.54050	6.20481
C	-0.06530	-4.57591	2.86884	H	-2.56400	0.78079	6.35339
C	-1.12895	-4.97428	3.59380	H	-3.08889	-0.87266	5.96287
C	-2.31482	-4.19275	3.81242	H	-3.71321	2.02485	4.29890
C	-2.46900	-2.89183	3.49242	H	-3.68177	1.46275	2.61204
Br	-4.42157	-2.37030	3.72081	H	-4.52641	0.50957	3.83388
C	-3.66171	1.14420	3.65457	H	-1.12914	-5.98506	3.98771
H	-1.31911	0.38994	-0.26538	H	-3.15780	-4.73748	4.22610
H	0.02027	-0.73396	-0.53522	H	0.74826	-5.26793	2.68059

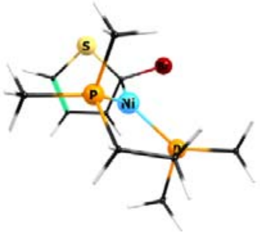

Geometry	**TS4Br**
E(Hatree)	-5574.54915
Gibbs(Hatree)	-5574.33023
Enthalpy(Hatree)	-5574.26416
Entropy Correction(Hatree)	-0.06529
# Imaginary Frequencies	-85.23

CARTESIAN COORDIANTES(ANGSTROEM)

RKS B3LYP def2-TZVP def2-TZVP/C RIJCOSX Grid4

P	-0.5737	2.026683	11.780562	H	-2.205835	3.795322	11.16648
Ni	-0.533779	-0.202868	11.64482	H	-2.848318	2.641928	12.36031
P	0.167332	-0.107589	9.522702	H	0.146448	-0.47904	7.073555
C	0.053657	3.184499	13.077863	H	-1.47363	-0.626014	7.793064
C	-2.248746	2.740793	11.454143	H	-0.309478	-1.936429	7.985548
C	0.400219	2.605392	10.292159	H	2.408788	-0.208314	8.468319
C	0.14714	1.710796	9.070809	H	2.111712	-1.574613	9.566277
C	-0.415415	-0.85183	7.934263	H	2.509551	0.006427	10.23001
C	1.973467	-0.500839	9.427731	C	-1.108205	-1.632617	12.85741
H	1.454752	2.555938	10.581118	S	-1.802398	-0.946701	14.33442
H	0.183109	3.65333	10.062058	C	-0.233695	-0.650403	14.99597
H	-0.84936	1.912003	8.665921	C	0.765611	-1.065726	14.1707
H	0.862796	1.928134	8.27159	C	0.299691	-1.659098	12.95259
H	-0.501532	3.01759	14.001784	Br	-2.188357	-3.050859	11.99431
H	-0.042135	4.234937	12.789215	H	-0.135023	-0.121713	15.93147
H	1.103736	2.963483	13.278051	H	1.814912	-0.964023	14.41553
H	-2.747027	2.176917	10.665817	H	0.919965	-2.294345	12.3367

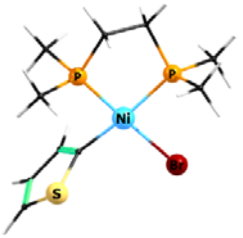

		Geometry			**9Br**	
		E(Hatree)			-5574.62951	
		Gibbs(Hatree)			-5574.33023	
		Enthalpy(Hatree)			-5574.26416	
		Entropy Correction(Hatree)			-0.06529	
		# Imaginary Frequencies			0.00	
CARTESIAN COORDIANTES(ANGSTROEM)						
RKS B3LYP def2-TZVP def2-TZVP/C RIJCOSX Grid4						
C	0.27556	0.056139	-0.248222	H	0.649838	3.126726	3.717436
S	0.067881	0.33079	1.44277	H	3.71744	2.415331	1.102876
C	1.752283	0.13064	1.799736	H	4.294794	3.784657	2.091172
C	2.41942	-0.129545	0.62424	H	5.036187	2.173859	2.253543
C	1.587051	-0.183188	-0.533011	H	5.621658	-0.987173	5.189945
Ni	2.503323	0.131084	3.577444	H	5.282553	-0.809614	6.92897
Br	2.007951	-2.179162	4.004572	H	4.367756	-1.982854	5.93189
P	2.871873	2.244222	3.349244	H	3.046904	0.474491	7.920543
C	3.568408	2.923355	4.936907	H	1.705354	1.026689	6.888799
C	4.449671	1.868583	5.613215	H	1.983173	-0.700236	7.091183
P	3.56625	0.239033	5.536637	H	4.703996	2.148542	6.638595
C	4.839676	-1.00086	5.949511	H	5.389465	1.74851	5.067862
C	1.460536	3.35582	3.025392	H	4.104544	3.85861	4.758821
C	4.10308	2.709837	2.078367	H	2.710613	3.16334	5.571987
C	2.478786	0.267631	7.011304	H	-0.564382	0.065554	-0.92432
H	1.760959	4.398074	3.152765	H	1.95466	-0.40392	-1.527094
H	1.09007	3.194733	2.013964	H	3.484557	-0.322345	0.580269

Supplementary Table 11. *DFT Optimized Geomerty Cartesian Coordinates.*

Supplementary References

1. Bach, I. *et al. Organometallics* **15,** 4959–4966 (1996).
2. SAINT. Version 8.34A Bruker AXS Inc., Madison, Wisconsin, USA. (1997-2013).
3. SADABS 2014/5 - Krause, L., Herbst-Irmer, R., Sheldrick, G. M. & Stalke, D. (2015). *J. Appl. Crystallogr.* **48**.
4. XT: Sheldrick, G. M.; *Acta Cryst*., **A**71, 3-8 (2015).
5. Least Squares function minimized:

   $\Sigma w(F_o^2-F_c^2)^2$
6. Standard deviation of an observation of unit weight:

   $[\Sigma w(F_o^2-F_c^2)^2/(N_o-N_v)]^{1/2}$

   where: N_o = number of observations

   N_v = number of variables

7. Cromer, D. T. & Waber, J. T.; *"International Tables for X-ray Crystallography"*, **Vol. IV**, The Kynoch Press, Birmingham, England, Table 2.2 A (1974).

8. Ibers, J. A. & Hamilton, W. C.; *Acta Crystallogr.*, **17**, 781 (1964).

9. Creagh, D. C. & McAuley, W.J .; *"International Tables for Crystallography"*, **Vol C**, (A.J.C. Wilson, ed.), Kluwer Academic Publishers, Boston, Table 4.2.6.8, pages 219-222 (1992).

10. Creagh, D. C. & Hubbell, J.H..; *"International Tables for Crystallography"*, **Vol C**, (A.J.C. Wilson, ed.), Kluwer Academic Publishers, Boston, Table 4.2.4.3, pages 200-206 (1992).

11. SHELXL-2016 Sheldrick, G. M.; *Acta Cryst.*, **C71**, 3-8 (2015).

12. OLEX2 – V1.2.8 Dolomanov, O.V.; Bourhis, L.J.; Gildea, R.J.; Howard, J.A.K.; Puschmann, H., OLEX2: *A complete structure solution, refinement and analysis program* (2009). *J. Appl. Cryst*., **42**, 339-341.

13. Frank Neese in ORCA – an ab initio, DFT, and Semiempirical Electronic Structure Package, version 3.0.3. The Max Planck Institute for Chemical Energy Conversion, Muelheim a. d. Ruhr, Germany, 2015.

14. [1] A.D. Becke, *J.Chem.Phys.* **98** (1993) 5648-5652 [2] C. Lee, W. Yang, R.G. *Parr, Phys. Rev. B* **37** (1988) 785-789 [3] S.H. Vosko, L. Wilk, M. Nusair, *Can. J. Phys.* **58** (1980) 1200-1211 [4] P.J. Stephens, F.J. Devlin, C.F. Chabalowski, M.J. Frisch, *J.Phys.Chem.* **98** (1994) 11623-11627.

15. A. Schaefer, C. Huber, and R. Ahlrichs, "Fully optimized contracted Gaussian-basis sets of triple zeta valence quality for atoms Li to Kr," *J. Chem. Phys.*, **100** (1994) 5829-35. DOI: 10.1063/1.467146.

16. Aleksandr V. Marenich, Christopher J. Cramer, and Donald G. Truhlar. *J. Phys. Chem.* **B**, 2009, 113 (18), pp 6378–6396

17. Y. Zhao and D. G. Truhlar, *Theor. Chem. Acc*., **120** (2008) 215-41. DOI: 10.1007/s00214-007-0310-x.

18. Becke A. D, *Phys Rev A Gen Phys*. 1988 Sep 15; 38(6):3098-3100.

19. *J. Chem. Phys*. 1993, **99**, 4597; *J. Chem .Phys*. 1998, **109**, 392.

20. *J. Chem. Phys*. 2009, 356, 98-109.

21. E. D. Glendening, C. R. Landis, and F. Weinhold, "NBO 6.0: Natural Bond Orbital Analysis Program," J. Comp. Chem. 34, 1429-1437 (2013).

22. Gaussian 09, Revision A.02, M. J. Frisch, G. W. Trucks, H. B. Schlegel, G. E. Scuseria, M. A. Robb, J. R. Cheeseman, G. Scalmani, V. Barone, G. A. Petersson, H. Nakatsuji, X. Li, M. Caricato, A. Marenich, J. Bloino, B. G. Janesko, R. Gomperts, B. Mennucci, H. P. Hratchian, J. V. Ortiz, A. F. Izmaylov, J. L. Sonnenberg, D. Williams-Young, F. Ding, F. Lipparini, F. Egidi, J. Goings, B. Peng, A. Petrone, T. Henderson, D. Ranasinghe, V. G. Zakrzewski, J. Gao, N. Rega, G. Zheng, W. Liang, M. Hada, M. Ehara, K. Toyota, R. Fukuda, J. Hasegawa, M. Ishida, T. Nakajima, Y. Honda, O. Kitao, H. Nakai, T. Vreven, K. Throssell, J. A. Montgomery, Jr., J. E. Peralta, F. Ogliaro, M. Bearpark, J. J. Heyd, E. Brothers, K. N. Kudin, V. N. Staroverov, T. Keith, R. Kobayashi, J. Normand, K. Raghavachari, A. Rendell, J. C. Burant, S. S. Iyengar, J. Tomasi, M. Cossi, J. M. Millam, M. Klene, C. Adamo, R. Cammi, J. W. Ochterski, R. L. Martin, K. Morokuma, O. Farkas, J. B. Foresman, and D. J. Fox, Gaussian, Inc., Wallingford CT, 2016.

23. Bader, R. F. W. A Quantum Theory of Molecular Structure and Its Applications.

24. Wagner, F. R., Bezugly, V., Kohout, M. & Grin, Y. Charge Decomposition Analysis of the Electron Localizability Indicator: A Bridge between the Orbital and Direct Space Representation of the Chemical Bond. *Chem. - A Eur. J.* **13,** 5724–5741 (2007).

25. Tian Lu, Feiwu Chen, J. Comp. Chem. 33, 580-592 (2012).

26. Webb, S. M. Phys. Scr. 2005, T115, 1011

27. Ravel, B.; Newville, M. J. Synchrotron Radiat. 2005, 12, 537.
